# Reciprocal regulation between nicotinamide adenine dinucleotide metabolism and abscisic acid and stress response pathways in Arabidopsis

**DOI:** 10.1371/journal.pgen.1008892

**Published:** 2020-06-22

**Authors:** Yechun Hong, Zhen Wang, Huazhong Shi, Juanjuan Yao, Xue Liu, Fuxing Wang, Liang Zeng, Zhi Xie, Jian-Kang Zhu

**Affiliations:** 1 Shanghai Center for Plant Stress Biology and Center for Excellence in Molecular Plant Sciences, Chinese Academy of Sciences, Shanghai, China; 2 University of Chinese Academy of Sciences, Shanghai, P.R. China; 3 Department of Chemistry and Biochemistry, Texas Tech University, Lubbock, Texas, United States of America; 4 Department of Horticulture and Landscape Architecture, Purdue University, West Lafayette, Indiana, United States of America; The University of North Carolina at Chapel Hill, UNITED STATES

## Abstract

Nicotinamide adenine dinucleotide (NAD) is an essential coenzyme that has emerged as a central hub linking redox equilibrium and signal transduction in living organisms. The homeostasis of NAD is required for plant growth, development, and adaption to environmental cues. In this study, we isolated a chilling hypersensitive *Arabidopsis thaliana* mutant named *qs-2* and identified the causal mutation in the gene encoding quinolinate synthase (QS) critical for NAD biosynthesis. The *qs-2* mutant is also hypersensitive to salt stress and abscisic acid (ABA) but resistant to drought stress. The *qs-2* mutant accumulates a reduced level of NAD and over-accumulates reactive oxygen species (ROS). The ABA-hypersensitivity of *qs-2* can be rescued by supplementation of NAD precursors and by mutations in the ABA signaling components SnRK2s or RBOHF. Furthermore, ABA-induced over-accumulation of ROS in the *qs-2* mutant is dependent on the SnRK2s and RBOHF. The expression of *QS* gene is repressed directly by ABI4, a transcription factor in the ABA response pathway. Together, our findings reveal an unexpected interplay between NAD biosynthesis and ABA and stress signaling, which is critical for our understanding of the regulation of plant growth and stress responses.

## Author summary

Nicotinamide adenine dinucleotide (NAD) is a coenzyme essential for metabolisms and signal transduction in all living organisms, but little is known about its role in ABA-mediated plant growth inhibition. Here we show that a mutation in *QS* gene that causes reduced level of NAD influences the ABA signal transduction in *Arabidopsis*, suggesting a possible conserved role of NAD in abiotic stress response in plants. In *Arabidopsis*, disruption of NAD homeostasis leads to ROS burst that results in a growth inhibition during exogenous ABA treatment. The ROS-mediated reduction of plant growth is fully restored by the mutation in genes encoding SnRK2 kinases, which are the core components in ABA signal transduction. A feedback repression of the *QS* transcription requires ABI4, which is a well-known transcription factor in the downstream of ABA signaling. Our study reveals the importance of NAD in ROS burst and plant adaption to environmental cues, and also provides insights into the unexpected interplay between NAD homeostasis and ABA-mediated plant growth inhibition.

## Introduction

Plants respond to harsh environments not only by activating protective stress responses but also by actively repressing growth. The interplay between plant growth and stress signaling pathways has been reported [1,2]. As a regulatory hub linking primary metabolism, redox regulation and energy signaling [3], nicotinamide adenine dinucleotide (NAD) is presumably an important factor for stress resistance and for plant growth regulation under stress environments [4,5]. However, the relationship between NAD metabolism and plant stress responses is poorly understood.

Abscisic acid (ABA) is a well-studied phytohormone that accumulates in response to abiotic stresses, such as water deficit and high salinity [6]. In higher plants, the *de novo* synthesis of ABA originates primarily from zeaxanthin [7–10]. The enzymatic step that catalyzes xanthoxin to ABA-aldehyde was elucidated by the discovery of the *ABA2* gene encoding a cytosolic short-chain dehydrogenase/reductase [11]. ABA2 is a key enzyme in the ABA biosynthesis pathway since the *aba2-1* mutant accumulates a remarkably reduced level of ABA [12]. In addition, the *aba2-1* mutant exhibits a phenotype of increased number of leaves [13]. In *Arabidopsis*, ABA is perceived by the PYRABACTIN RESISTANCE1 (PYR1)/PYR1-LIKE (PYL) / REGULATORY COMPONENTS OF ABA RECEPTOR (RCAR) family of receptor proteins and clade A protein phosphatase of type 2C (PP2C) co-receptors. ABA triggers the association of these two receptor components, which leads to inactivation of the PP2C and activation of the PP2C-repressed sucrose non-fermenting-1 (SNF1)-related protein kinase 2s (SnRK2s). The activation of SnRK2s is required for the downstream responses of ABA [14]. The SnRK2s directly phosphorylate and activate the ABA insensitive 4 (ABI4), an AP2-type transcription factor, and other transcription factors including basic region/leucine zipper (bZIP)-type transcriptional factors positively regulating the ABA response [15–17]. In addition to its role in stress response, ABA also plays an important role in plant growth and development through modulating the kinase activity of SnRK2s. The triple knockout mutant of the three kinases, *snrk2*.*2/2*.*3/2*.*6*, shows increased leaf emergence and seedling growth, suggesting that the ABA signaling mediated by the SnRK2s is important for plant growth [13].

Reactive oxygen species (ROS) function as second messengers to positively regulate ABA signaling [18]. An important source of ROS production in plants is the plasma membrane-localized NAD(P)H oxidases [19,20]. Previous studies have shown that one of the ten NAD(P)H oxidases in *Arabidopsis*, RBOHF, is substrate of SnRK2s and responsible for ABA-induced ROS production [21]. The ROS burst in response to ABA is required for ABA-mediated stomatal movements, root growth, seed dormancy and seed germination [22–24]. Furthermore, the PP2Cs are often considered as the targets of ROS in modulating ABA responses [25–27]. Dysfunction of *ABI1* causes increased shoot and bud growth in poplars [28], consistent with the ABA-induced ROS regulating developmental processes. There is overwhelming evidence showing that cellular ROS plays vital roles in plant growth, development and response to stress conditions [29].

NAD is known as an ubiquitous coenzyme essential for cellular metabolism and other processes, including redox equilibrium, cellular energy, DNA repair, calcium-dependent signaling, and lifespan extension in eukaryotes [30–32]. The homeostasis of NAD maintained by its biosynthesis and catabolism is required for proper cellular functions. In *Arabidopsis*, chloroplast L-aspartate serves as the precursor of NAD in the *de novo* biosynthesis pathway [33]. In plastids, the formation of quinolinate is catalyzed by aspartate oxidase (AO) and quinolinate synthase (QS), and quinolinate is rapidly converted to nicotinate mononucleotide (NaMN) by quinolinate phosphoribosyltransferase (QPT). NaMN is adenylated in cytoplasm to produce nicotinate adenine dinucleotide (NaAD), which is converted to NAD through amidation [4,32]. The activity of QS is dependent on the oxygen-sensitive Fe-S cluster reconstituted through its Cys desulfurase domain [34]. Moreover, land plants also evolved a salvage pathway that stabilizes the cellular NAD pool. In this pathway, NIM, a metabolite of NAD, is initially converted by nicotinamidase into nicotinate (NA), which is used as the substrate in the three step Preiss-Handler pathway to generate NAD [35,36]. The salvage pathway is important in plant abiotic stress resistance [4]. Blocking the salvage pathway through the loss-of-function of *NIC1* leads to a hypersensitive phenotype to high salt and ABA treatments [36,37]. Application of nicotinamide reduces H_2_O_2_ accumulation and inhibits ABA-induced stomatal closure, and NAD is likely salvaged from nicotinamide during seed germination [38,39]. To date no null mutants of the genes in the *de novo* NAD biosynthesis pathway have been identified, indicating an essential role of the *de novo* pathway in plant growth and development [40]. The *old5* mutation in QS enzyme in the *de novo* NAD biosynthesis pathway decreases the Cys desulfurase activity of the QS enzyme and increases the steady state levels of NAD, which coincides with increased expression of oxidative stress marker genes and an early onset of senescence [41]. A recent report suggested that NAD is required for the biosynthesis of ABA and proline and plant response to salt stress [42]. However, the role of NAD synthesis in plant response to ABA has remained largely unknown.

Here we isolated an *Arabidopsis* mutant hypersensitive to chilling stress, *htc1*. A point mutation in the region encoding the NadA domain of the *QS* gene was identified through map-based cloning and whole-genome resequencing, and this mutant was subsequently renamed as *qs-2*. The mutant has dramatically decreased levels of NAD and its derivatives. The *qs-2* mutant also displays hypersensitivity to exogenously applied ABA. ABA treatment results in over-accumulation of ROS and a further impaired growth of *qs-2* mutant, which can be suppressed by the *snrk2*.*2/2*.*3/2*.*6* mutations. We show that the expression of *QS* gene is repressed directly by ABI4 through its binding to the *QS* promoter. Our findings reveal not only a critical role of NAD in ABA and stress signaling, but also how ABA and stress may control plant growth by modulating NAD biosynthesis.

## Results

### The *qs-2* mutation causes chilling hypersensitivity

**Fig 1:**
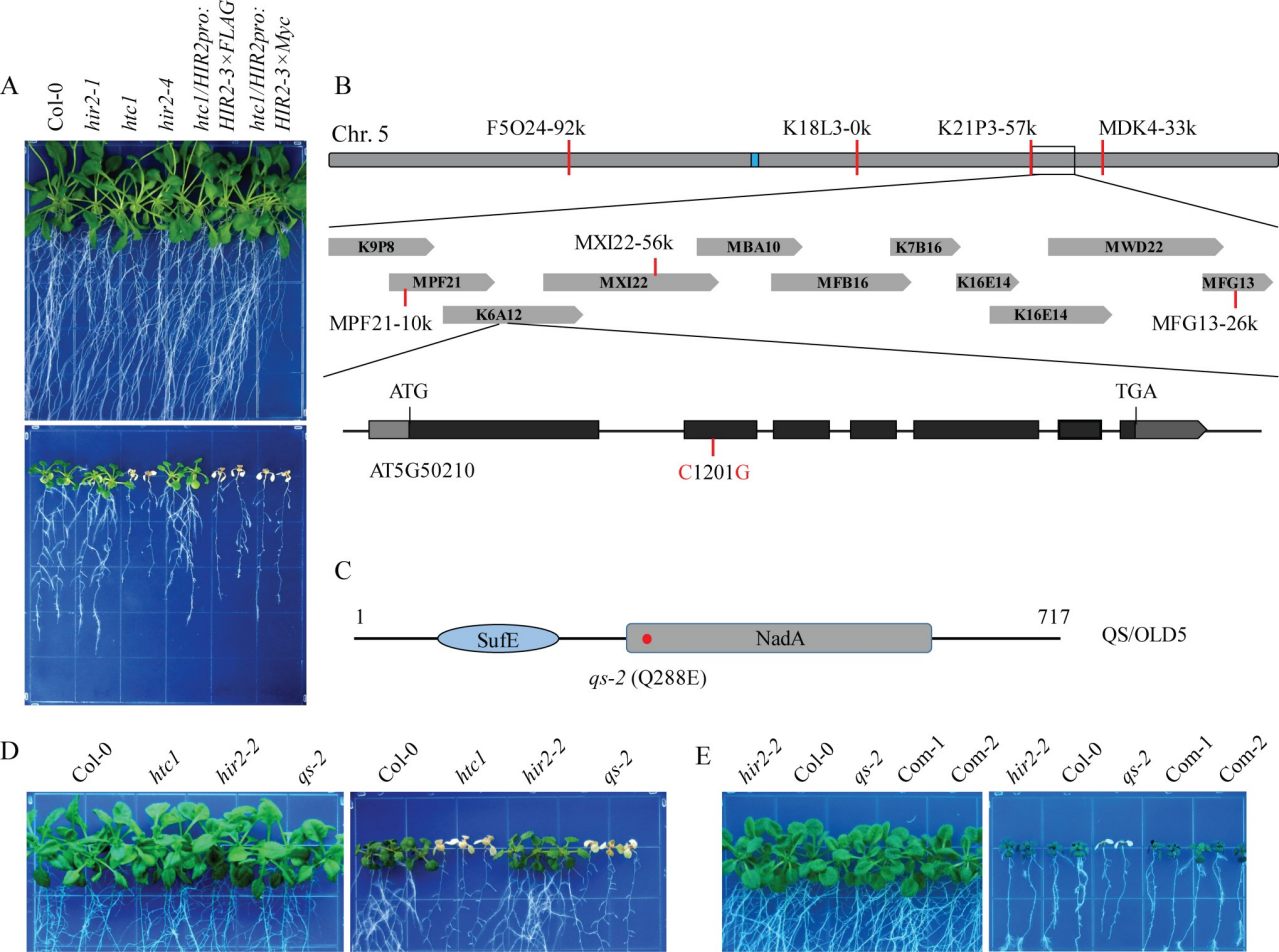
Identification of the *HTC1*/*QS* gene. (A) The *htc1* mutant plants displayed hypersensitivity to chilling stress. Seven-day-old seedlings of Col-0, *hir2-1*, *htc1*, *hir2-4*, and two transgenic lines grown on 1/2 MS medium plates were transferred to a growth chamber at 22°C (upper) or 4°C (bottom) for an additional 21 days. (B) Map-based cloning of the *qs-2* mutation. A total of 96 samples were used for rough genetic mapping which narrowed the *htc1* mutation to the region between the BAC clones MPF21-10k and MXI22-56k on chromosome 5. An C to G mutation in the second exon of AT5G50210 was identified by genome re-sequencing. (C) The structure of QS protein. SufE, Fe-S metabolism associated domain; NadA, quinolinate synthase domain. The position of the Q288E substitution in the *qs-2* mutant is indicated by a red dot. (D) Chilling sensitivity of the *qs-2* mutant. Seven-day-old seedlings of Col-0 wild-type, *htc1* and its segregated alleles *hir2-2*, *qs-2* grown on 1/2 MS medium plates at 22°C (left) or 4°C (right) for 21 days. (E) Molecular complementation assay of the chilling hypersensitive phenotype of *qs-2* mutant. Seven-day-old seedlings of Col-0 wild-type, *qs-2* and two complementation lines were grown on 1/2 MS medium at 22°C (left) or 4°C (right) for 21 days.

Membrane proteins, including ion channels, transporters and membrane-anchored receptors, play important roles in plant abiotic stress responses [43]. To identify membrane proteins critical for the regulation of plant cold tolerance, we screened a mutant pool containing 510 T-DNA insertion mutants of genes encoding membrane proteins, which was obtained from the Arabidopsis Biological Resource Center (ABRC). One of the chilling hypersensitive mutants was isolated from the mutant line SALK_124393C and was designed as *hypersensitive to chilling stress 1* (*htc1*). The SALK_124393C line harbors a T-DNA insertion in the *HIR2* (*HYPERSENSITIVE INDUCED REACTION 2*) gene, which is a pathogen inducible gene and plays a role in effector-triggered immunity [44]. We performed a molecular complementation assay to determine whether the mutation in *HIR2* is the causal mutation, and found that either *HIR2-3×FLAG* or *HIR2-3×Myc* driven by the native promoter failed to rescue the chilling hypersensitive phenotype of the *htc1* mutant (Fig 1A). In addition, two null mutant alleles of *HIR2*, *hir2-1* and *hir2-4*, showed a growth phenotype similar as the wild type under chilling stress (Fig 1A). These results indicated that the chilling hypersensitivity of the *htc1* mutant is not caused by the T-DNA knockout mutation in the *HIR2* gene. We thus performed map-based cloning to identify the causal mutation for the chilling hypersensitive phenotype of the *htc1* mutant. Genetic mapping located the *htc1* mutation in the ~1 Mb region between the BAC clones MPF21-10k and MXI22-56k on chromosome 5 (Fig 1B). We then performed whole-genome resequencing of the *htc1* mutant. Ten mutations were identified in this region but only the mutation of C1201G, a single nucleotide substitution in the second exon of AT5G50210, results in an amino acid substitution of Q288E (Fig 1B and S1 Table). AT5G50210 encodes the quinolinate synthase (QS, also known as OLD5) involved in the *de novo* biosynthesis of NAD. The QS enzyme contains SufE and quinolinate synthase (NadA) domains, and the Q288E substitution is in the NadA domain (Fig 1C and S1A Fig). The mutant harboring C1201G in the *QS* gene was segregated away from the T-DNA insertion of *HIR2* and was renamed as *qs-2*. The *qs-2* mutant showed a chilling hypersensitive phenotype same as the *htc1* mutant, and the *qs-2* mutation did not alter the transcription level of *QS* gene (Fig 1D and S1B Fig). We performed molecular complementation assay by introducing *QSpro*:*QS-3×FLAG* into the *qs-2* mutant, and two tested transgenic lines, named as Com-1 and Com-2, showed the expression of the QS-3×FLAG fusion protein (S1C Fig). A chilling tolerance assay showed that the expression of the wild type QS gene fully restored the chilling tolerance of the *qs-2* mutant to the wild type level (Fig 1E). These results showed that the single nucleotide mutation causing Q288E substitution in the QS enzyme is responsible for the chilling hypersensitivity of the *qs-2* mutant.

### QS functions in NAD biosynthesis in chloroplasts

**Fig 2:**
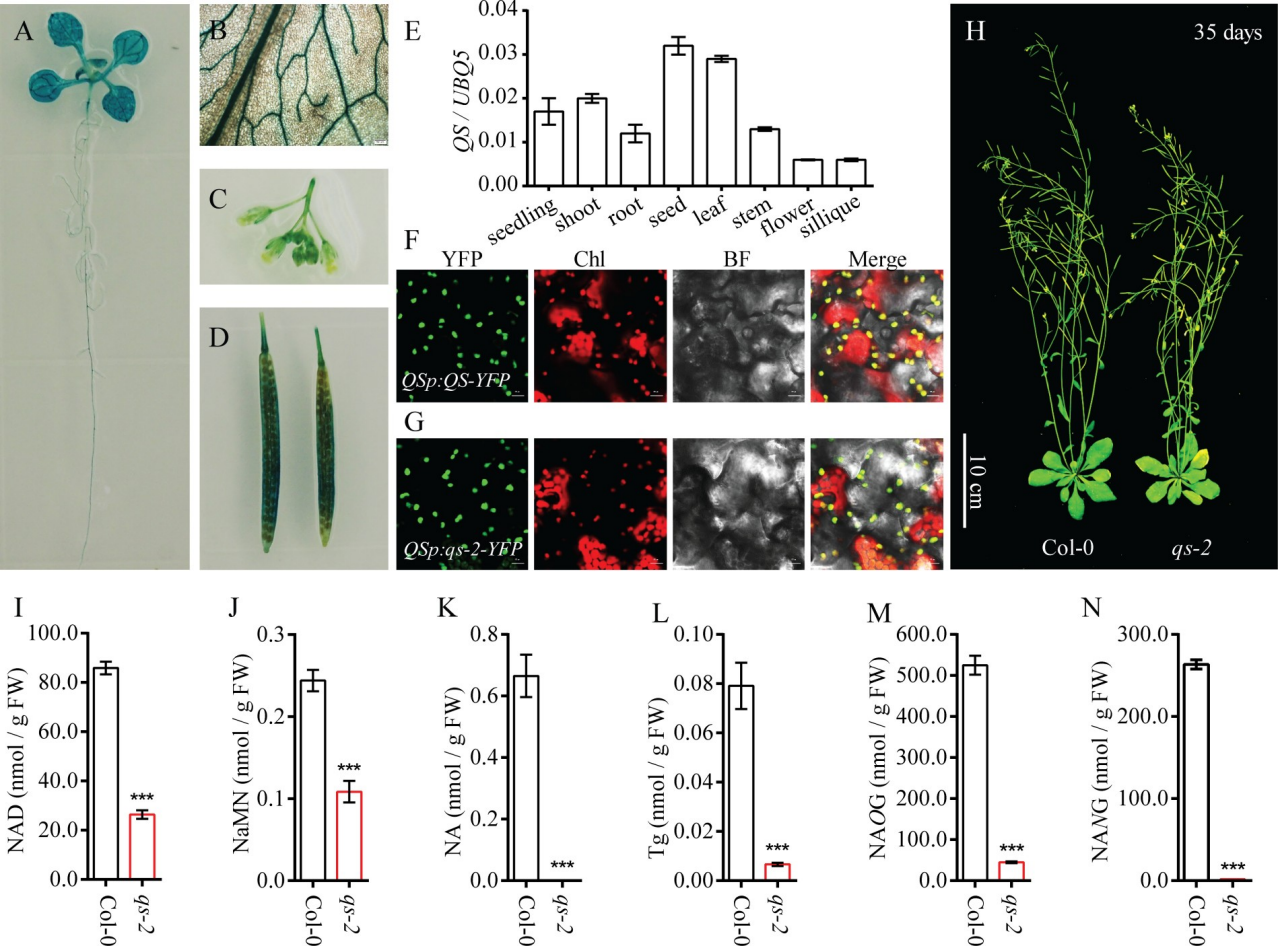
Characterization of the *QS* gene in *Arabidopsis*. (A—D) The expression pattern of the *QS* gene analyzed by promoter-GUS analysis. Histochemical staining of 10-day-old seedling (A), rosette leaf (B), flower (C) and silique (D) of the *QSpro*:*QS-GUS* transgenic plants. (E) The expression of *QS* in different tissues determined by qRT-PCR. Values are means ± SD (n = 3). (F, G) Subcellular localization of QS-YFP and *qs-2*-YFP fusion proteins. The YFP signals in the transgenic plants harboring the *QSpro*:*QS-YFP* (F) and *QSpro*:*qs-2-YFP* (G) were observed using confocal microscopy. YFP, yellow fluorescence protein. Chl, chlorophyll autofluorescence. BF, bright field. Bars, 10 μm. (H) Growth phenotypes of Col-0 and *qs-2* mutant plants in soil for 35 days. Bars, 10 cm. (I—N) Analysis of the metabolites in the rosette leaves of Col-0 and *qs-2* mutants. The contents of NAD (I), NaMN (J), NA (K), Tg (L), NA*O*G (M), and NA*N*G (N) were determined in the rosette leaves of Col-0 and *qs-2* plants grown in 1/2 MS for 3 weeks. The data represent the means ± SD (n = 5). Asterisks indicate significant differences from the Col-0 plants, *** *P* < 0.001, Student’s t-test.

To study the expression pattern of *QS* gene in *Arabidopsis*, we generated the transgenic plants harboring *QSpro*:*QS-GUS*. Histochemical staining showed that the GUS activity could be detected in all the tissues tested, including whole seedlings, rosette leaves, flowers and siliques, and the GUS activity was high in the veins of leaves (Fig 2A–2D). We also examined *QS* transcript levels in different tissues using quantitative real-time PCR (qRT-PCR), and the result showed that the *QS* gene is expressed in various tissues with relatively high expression levels in seeds and leaves (Fig 2E). To determine whether the mutation in QS affects its localization, we amplified the *QS* gene from wild type (Col-0) and the *qs-2* mutant and generated constructs containing the wild type *QS* (*QSpro*:*QS-YFP*) and the mutated *QS* (*QSpro*:*qs-2-YFP*), respectively. The transgenic plants harboring these constructs displayed YFP signals only in the chloroplasts (Fig 2F and 2G), indicating that the Q288E substitution has no effect on the chloroplast localization of QS. A recent study showed that a C to T change in the first exon encoding the SufE domain of QS in the *old5* mutant caused early leaf senescence [41]. We thus determined whether the *qs-2* mutation in the NadA domain of QS could also result in an early senescence phenotype. Both 10-day-old and 35-day-old *qs-2* plants had leaves more yellowish than the Col-0 wild-type (Fig 2H and S1D Fig). Further analysis verified that *qs-2* plants possess less chlorophyll b resulting in lower total chlorophyll content than Col-0 wild type (S1E–S1G Fig). These results indicate that the NadA domain of the chloroplast-localized QS is also important for plant growth and development. Since QS is a critical enzyme for NAD biosynthesis, we compared the contents of NAD and its intermediates in the *qs-2* mutant and wild type plants. The NAD content in the leaves of *qs-2* mutant was clearly reduced compared with that in the wild type (Fig 2I). In addition, the intermediates for the NAD biosynthesis, including NaMN, NA, and NA conjugates Tg (trigonelline), NA*O*G (nicotinate *O*-glucoside) and NA*N*G (nicotinate *N*-glucoside), showed significant lower levels in the *qs-2* mutant than in the wild type plants (Fig 2J–2N). Since the ratio of NAD/NADH is important in cellular metabolisms and redox reactions, we determined the contents of both oxidized and reduced forms of pyridine nucleotide. The *qs-2* mutant had significantly increased NADH while decreased NAD level when compared with wild type, resulting in a notably lower NAD/NADH ratio in the mutant than in the wild-type plants (S2 Fig). These results support that the QS enzyme is critical for the steady state of NAD and the homeostasis of NAD/NADH in *Arabidopsis*.

### The *qs-2* mutant is hypersensitive to ABA and salt stress but resistant to drought stress

**Fig 3:**
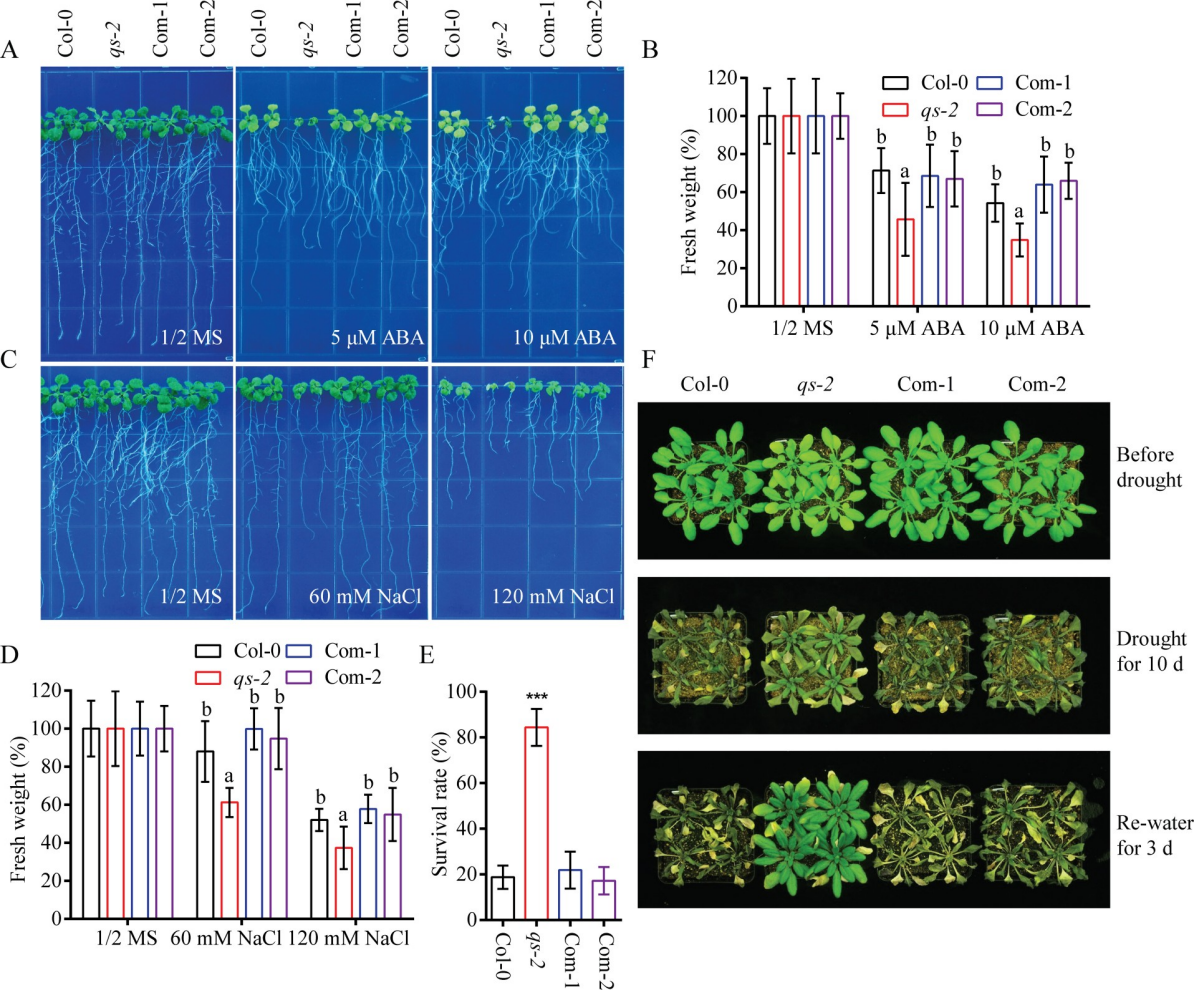
Abiotic stress responses of the *qs-2* mutant. (A) Phenotype of the *qs-2* mutant under exogenous ABA treatment. Five-day-old seedlings of Col-0, *qs-2* and two complementation lines grown on 1/2 MS supplemented with 0, 5 or 10 μM ABA for 8 days. (B) The fresh weight of the seedlings shown in (A). Values are the means ± SD of 3 replicates, and each replicate included 9 plants per genotype. The letters a and b above the columns indicate significant difference relative to Col-0 and *qs-2* mutant, respectively (*P* < 0.05, Student’s t-test). (C) Salt sensitivity of the *qs-2* mutant. Five-day-old seedlings of Col-0 wild-type, *qs-2* and two complementation lines grown on 1/2 MS medium containing 0, 60 or 120 mM NaCl for 8 days. (D) The fresh weight of the seedlings after salt treatment for 8 days. Values are means ± SD (n = 3, each replicate contained 9 plants per genotype). The letters a and b above the columns indicate significant difference relative to Col-0 and *qs-2* mutant, respectively (*P* < 0.05, Student’s t-test). (E) The survival rate of Col-0, *qs-2*, Com-1, and Com-2 plants shown in (F). Values indicate means ± SD (n = 3 biological replicates, 16 plants per genotype for each replicate). *** *P* < 0.001, Student’s t-test. (F) Drought resistance assay of Col-0, *qs-2*, Com-1, and Com-2 plants. Twenty-one-day-old plants were subjected to drought stress for 10 days, and then watered for 3 days for recovery.

Several studies reported that NAD plays important roles in plant response to environment cues [40,45]. We thus examined the response of *qs-2* mutant to ABA and different abiotic stresses. The *qs-2* mutant displayed a larger decrease in fresh weight compared to the Col-0 wild type under ABA treatment, and the ABA-hypersensitivity phenotype was suppressed in the complementation lines (Fig 3A and 3B). Salt tolerance assay showed that the *qs-2* mutant was less tolerant to salt stress, while the complementation lines displayed a similar level of salt tolerance as the wild type (Fig 3C and 3D). The result suggests that a proper NAD level is required for salt tolerance in *Arabidopsis*. Interestingly, the *qs-2* mutant was more resistant to drought stress than the wild type, and the complementation lines rescued the drought resistance phenotype of *qs-2* (Fig 3E and 3F). Altogether, these results suggest that NAD homeostasis is critical for plant abiotic stress response.

### The NAD precursors rescue the ABA-hypersensitivity of *qs-2* mutant plants

**Fig 4:**
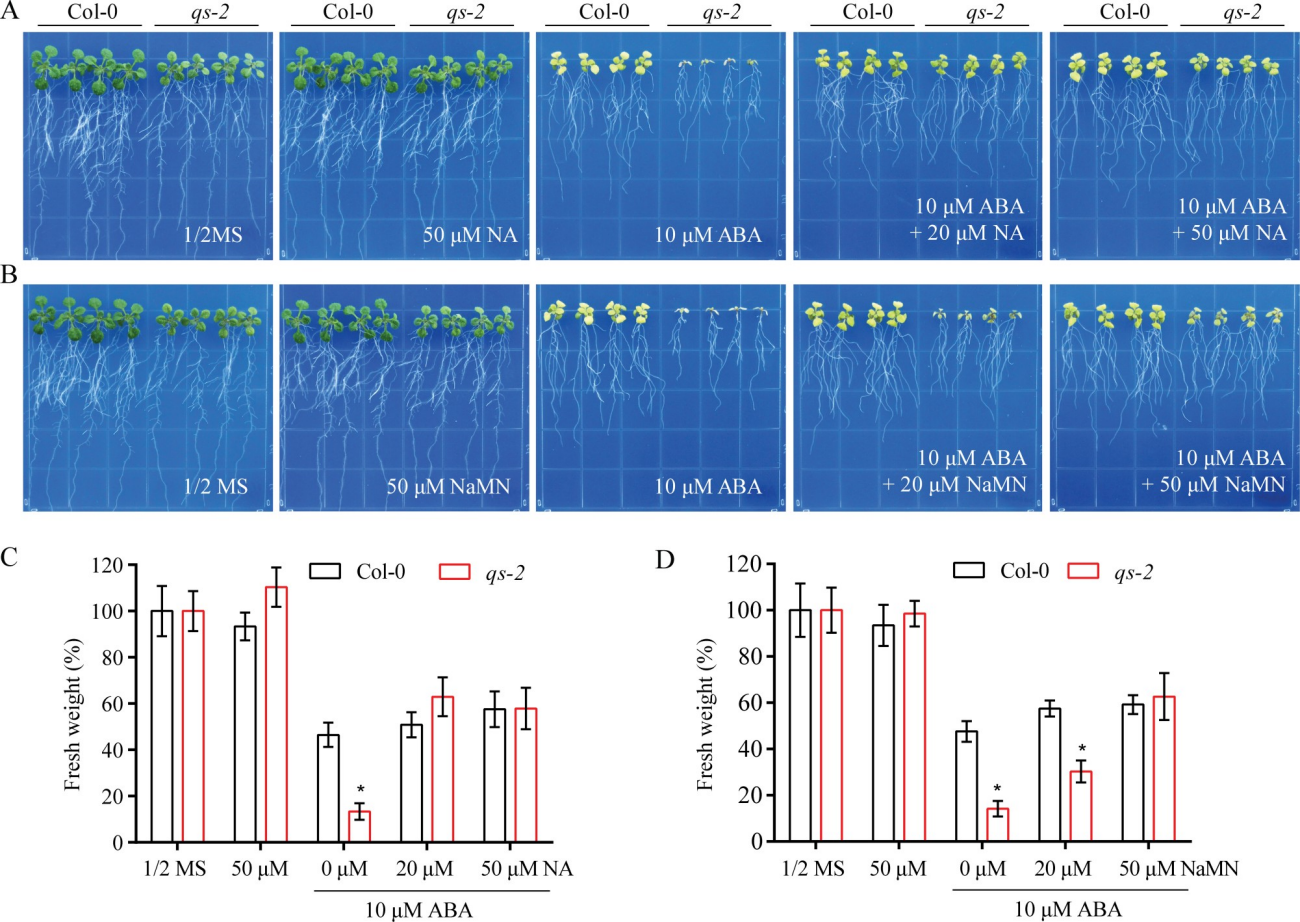
The NAD intermediates rescue the ABA-hypersensitive phenotype of *qs-2* mutant. (A, B) ABA sensitivity of the *qs-2* mutant without or with NA (A) or NaMN (B). Five-day-old seedlings of Col-0 and *qs-2* were grown on 1/2 MS medium supplemented with 10 μM ABA and different concentrations of NA (A) or NaMN (B) for 8 days. (C, D) The relative fresh weight of the seedlings shown in (A) and (B). Values are means ± SD of 3 replicates, each replicate contained 9 plants per genotype. * *P* < 0.05, Student’s t-test.

It is possible that the NAD deficiency in the *qs-2* mutant is the cause of ABA-hypersensitivity of the mutant. We tested whether exogenous application of the NAD precursors could rescue the *qs-2* phenotypes. It has been shown that supplementation of NA or NA conjugates rescues the lethality of the null mutant of *QS* through activating the NAD salvage pathway [40]. We therefore tested the response of *qs-2* mutant and wild type plants to ABA in the presence of different concentrations of NaMN or NA. After treatment for 8 days, the inhibition of *qs-2* mutant by ABA was clearly alleviated by the addition of NA or NaMN, and the sensitivity of the *qs-2* mutant to ABA was rescued to the wild type level by 20 or 50 μM NA, or 50 μM NaMN (Fig 4A–4D). These results strongly support that the deficiency of NAD is responsible for the ABA-hypersensitivity of *qs-2*. It was recently reported that NAD is involved in plant response to salt stress by increasing stress-induced ABA accumulation [42]. We determined the ABA contents in the wild-type and *qs-2* mutant under normal growth conditions. The ABA content in the leaves of *qs-2* mutant was significantly lower than that in the wild type, and the reduction of ABA content in the *qs-2* mutant was alleviated by supplement of 50 μM NaMN or NA (S3A Fig). This result further supports that the NAD level plays a critical role in maintaining ABA levels in plants. The effect of ABA level on the ABA-hypersensitivity of the *qs-2* mutant was assessed by analyzing the *aba2-1*, which is defective in ABA biosynthesis and known to have a reduced ABA level [12], and the *qs-2aba2-1* double mutant. The result showed that the *aba2-1* mutant was not hypersensitive to ABA and the double mutant behaved like the *qs-2* single mutant (S3B and S3C Fig). This result suggests that the hypersensitivity of *qs-2* to ABA is not a consequence of the reduced ABA level but is caused by the reduced NAD level in the *qs-2* mutant.

### SnRK2s mediate the ABA-hypersensitivity of the *qs-2* mutant

**Fig 5:**
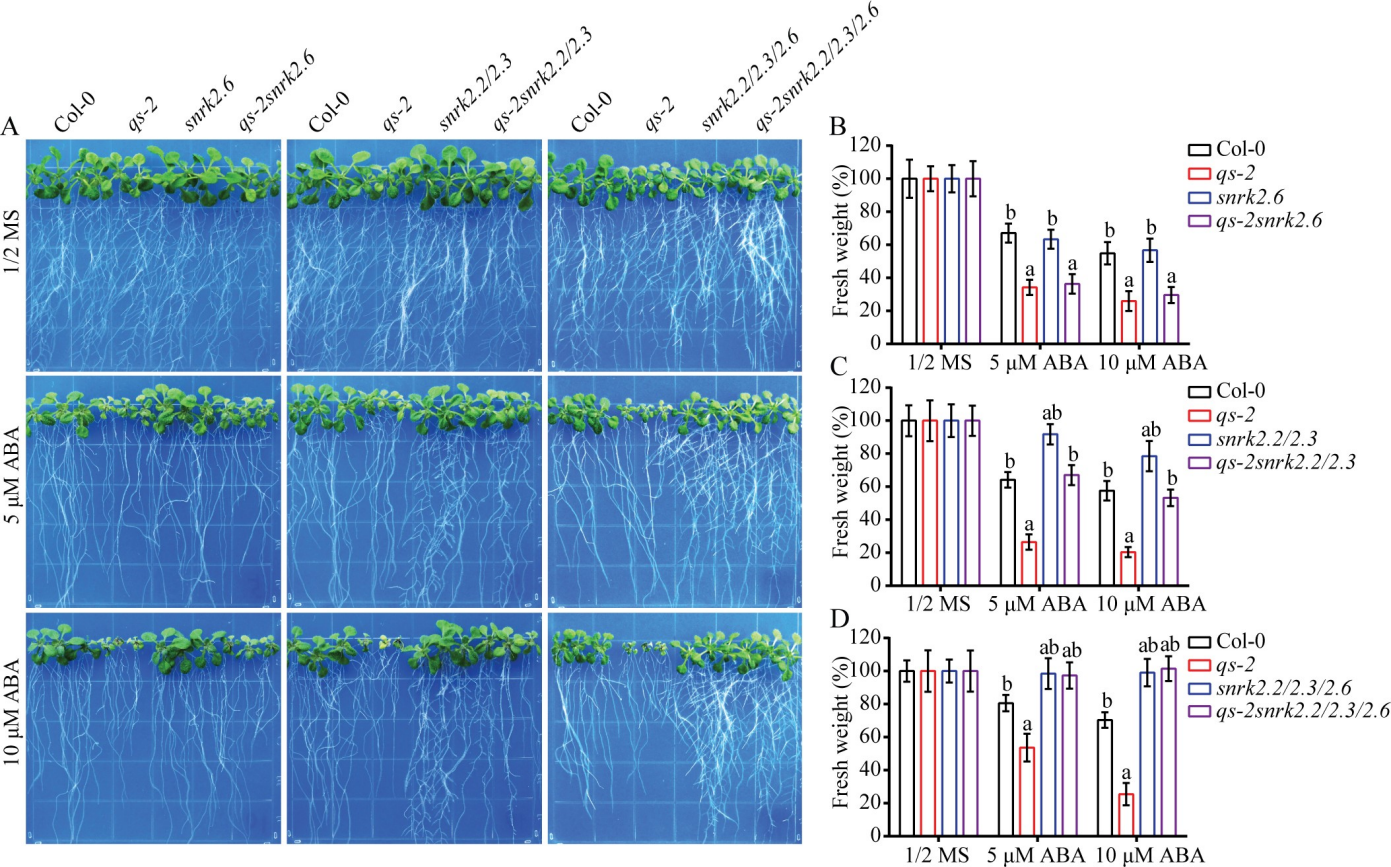
Genetic interaction between *QS* and *SnRK2s* in plant growth response to ABA. (A) Genetic relationship between *qs-2* and *SnRK2*.*2/2*.*3/2*.*6* mutations in response to ABA treatment. The images shown were 5-day-old Col-0, *qs-2*, *snrk2*.*6*, *qs-2snrk2*.*6*, *snrk2*.*2/2*.*3*, *qs-2snrk2*.*2/2*.*3*, *snrk2*.*2/2*.*3/2*.*6*, and *qs-2snrk2*.*2/2*.*3/2*.*6* seedlings grown on 1/2 MS plates supplemented with 0, 5 or 10 μM ABA for 8 days. (B—D) The fresh weight of the seedlings shown in (A). Values are means ± SD of 3 replicates, and each replicate contained 9 plants per line. The letters a and b above the columns indicate significant difference relative to Col-0 and *qs-2* mutant, respectively (*P* < 0.05, Student’s t-test).

We attempted to explore the molecular mechanism underlying the ABA-hypersensitivity of the *qs-2* mutant by using the mutants of SnRK2s, critical components in the ABA signaling pathway. The *qs-2snrk2*.*6*, *qs-2snrk2*.*2/2*.*3*, and *qs-2snrk2*.*2/2*.*3/2*.*6* mutants were generated by crossing *qs-2* with *snrk2*.*2/2*.*3/2*.*6* (S4 Fig). Five-day-old seedlings of the wild-type, *qs-2*, *snrk2*.*6*, *qs-2snrk2*.*6*, *snrk2*.*2/2*.*3*, *qs-2snrk2*.*2/2*.*3*, *snrk2*.*2/2*.*3/2*.*6*, and *qs-2snrk2*.*2/2*.*3/2*.*6* were grown for 8 days on the medium containing different concentrations of ABA to test their growth response (Fig 5A). Although the *snrk2*.*6* single mutant did not significantly affect the ABA-hypersensitivity of *qs-2* (Fig 5B), the *snrk2*.*2/2*.*3* double mutations clearly rescued the growth of the *qs-2* mutant (Fig 5C). The *qs-2snrk2*.*2/2*.*3/2*.*6* mutant behaved like the *snrk2*.*2/2*.*3/2*.*6* triple mutant, which showed insensitivity to ABA treatment (Fig 5D). These results show that the *snrk2* mutations are epistatic of *qs-2* in terms of ABA response and suggest that the QS enzyme and likely its product NAD act upstream of SnRK2s in the ABA response pathway.

### The ABA-hypersensitivity and ROS over-accumulation of *qs-2* mutant plants are dependent on the plasma membrane NADPH oxidase RBOHF

**Fig 6:**
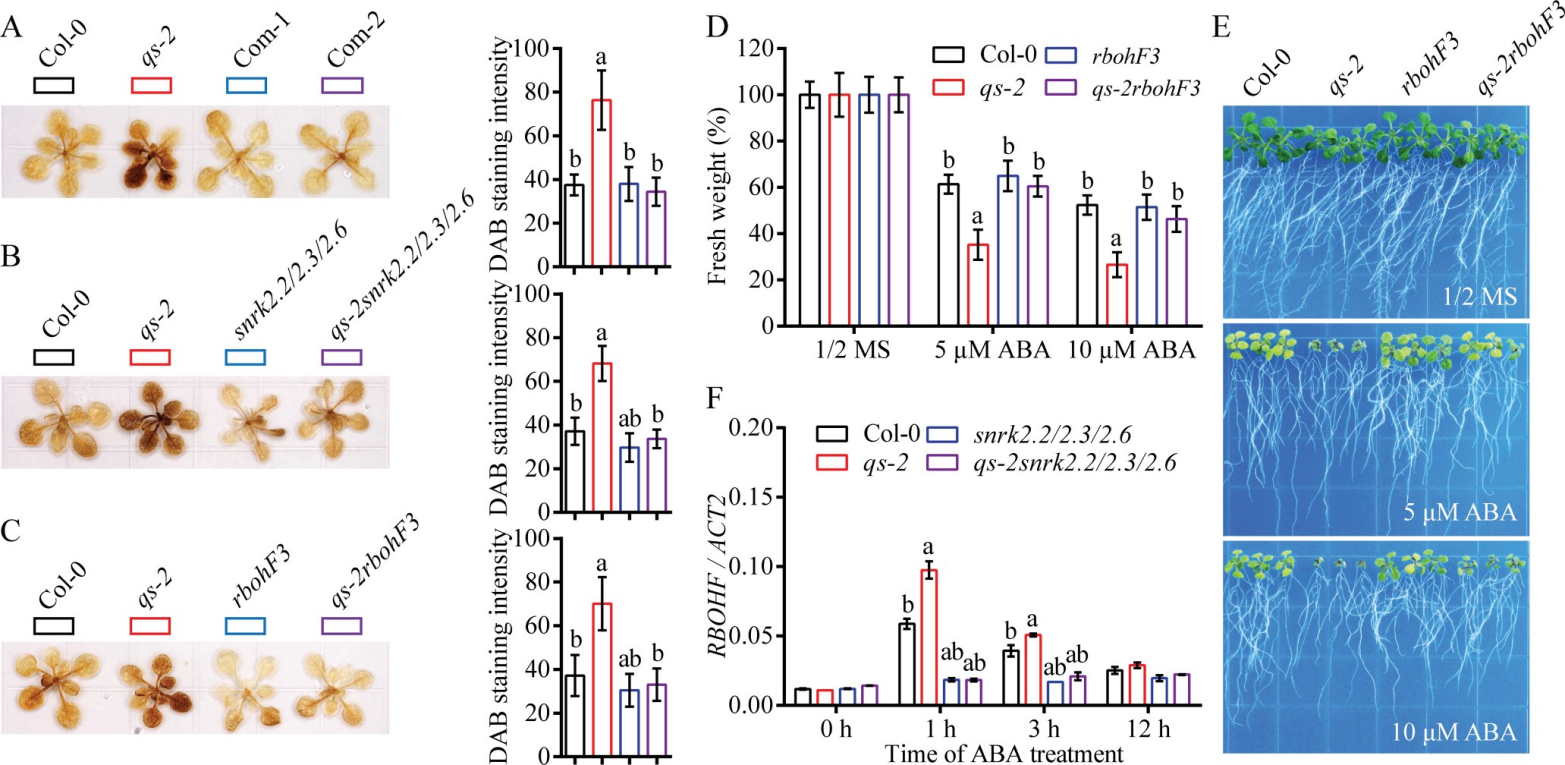
ROS over-accumulation in the *qs-2* mutant requires SnRK2s and RBOHF. (A—C) DAB staining showing H_2_O_2_ accumulation in the rosette leaves. Twenty-day-old Col-0, *qs-2*, Com-1 and Com-2 plants after ABA treatment for 1 day (A). Twenty-day-old Col-0, *qs-2*, *snrk2*.*2/2*.*3/2*.*6*, and *qs-2snrk2*.*2/2*.*3/2*.*6* plants were subjected to 10 μM ABA treatment for 1 day before DAB staining (B). Twenty-day-old Col-0 wild-type, *qs-2*, *rbohF3*, *qs-2rbohF3* plants after 10 μM ABA treatment for 1 day (C). The DAB staining intensity as determined by ImageJ. The letters a and b above the columns indicate significant difference relative to Col-0 and *qs-2* mutant, respectively (n = 5 plants, six leaves per plant, *P* < 0.05, Student’s t-test). (D) Genetic relationship between *qs-2* and *rbohF* mutations. Five-day-old seedlings of Col-0, *qs-2*, *rbohF3*, and *qs-2rbohF3* were grown on 1/2 MS supplemented with 0, 5 or 10 μM ABA for 8 days. (E) The fresh weight of the seedlings shown in (D). Values are means ± SD (n = 3 biological replicates, and each replicate contained 9 plants per genotype). The letters a and b above the columns indicate significant difference relative to Col-0 and *qs-2* mutant, respectively (*P* < 0.05, Student’s t-test). (F) The transcript levels of *RBOHF* determined by qRT-PCR in 10-day-old Col-0, *qs-2*, *snrk2*.*2/2*.*3/2*.*6*, *qs-2snrk2*.*2/2*.*3/2*.*6* plants after ABA treatment for the indicated hours. The letters a and b above the columns indicate significant difference relative to Col-0 and *qs-2* mutant, respectively (n = 3, *P* < 0.05, Student’s t-test). *ACT2* was used as an internal control.

Disruption of NAD homeostasis induces reactive oxygen species (ROS) and inhibits plant growth [41,46]. Since the NAD level is markedly reduced in the *qs-2* mutant (Fig 2I), we examined whether the *qs-2* mutant accumulates more ROS. The hydrogen peroxide (H_2_O_2_) level detected by 3,3’-diaminobenzidine (DAB) staining showed that the leaves of *qs-2* mutant accumulated H_2_O_2_ to higher levels than the wild type leaves, and the ROS was restored to the wild type level in the complementation lines (Fig 6A). This result indicates that reduced NAD levels promote ROS accumulation in *Arabidopsis*. Because the *snrk2* mutations rescued the ABA-hypersensitive phenotype of *qs-2* mutant (Fig 5), we further tested whether the *snrk2* mutations could suppress the ROS over-accumulation phenotype of the *qs-2* mutant. As shown in Fig 6B, the triple mutations of *snrk2*.*2/2*.*3/2*.*6* clearly recovered the ROS content of the *qs-2* mutant to the wild type level, indicating that the over-accumulation of ROS in *qs-2* mutant is mediated by the SnRK2s. To pinpoint the source of ROS over-accumulation in the *qs-2* mutant, we first tested whether the plasma membrane NADPH oxidase, which is a downstream target of SnRK2s and generates apoplastic ROS in response to stress [21], are required for the over-accumulation of ROS in the *qs-2* mutant. We generated the *qs-2rbohF3* double mutant, and ROS staining showed that the H_2_O_2_ level in the *qs-2rbohF3* double mutant was clearly lower than that in the *qs-2* single mutant under ABA treatment (Fig 6C), indicating that the plasma membrane NADPH oxidase RBOHF is required for the over-accumulation of ROS in the *qs-2* mutant in response to ABA. Furthermore, like the *snrk2* mutant, the *rbohF3* mutation suppressed the ABA-hypersensitivity of the *qs-2* mutant (Fig 6D and 6E), further supporting that the plasma membrane NADPH oxidase RBOHF mediates the effects of the *qs-2* mutation on ROS accumulation in *Arabidopsis*. In addition, we analyzed the transcript levels of *RBOHF* in Col-0, *qs-2*, *snrk2*.*2/2*.*3/2*.*6* and *qs-2snrk2*.*2/2*.*3/2*.*6* plants in response to ABA treatment. The transcript level of *RBOHF* was highly upregulated by ABA and the upregulation was enhanced by the *qs-2* mutation. However, the upregulation in the wild type and the ABA-enhanced upregulation in the *qs-2* mutant was abolished by the *snrk2*.*2/2*.*3/2*.*6* triple mutations (Fig 6F). These results showed that the induction of *RBOHF* expression by ABA requires the SnRK2s.

### The transcription factor ABI4 binds to the *QS* promoter and represses *QS* transcription

**Fig 7:**
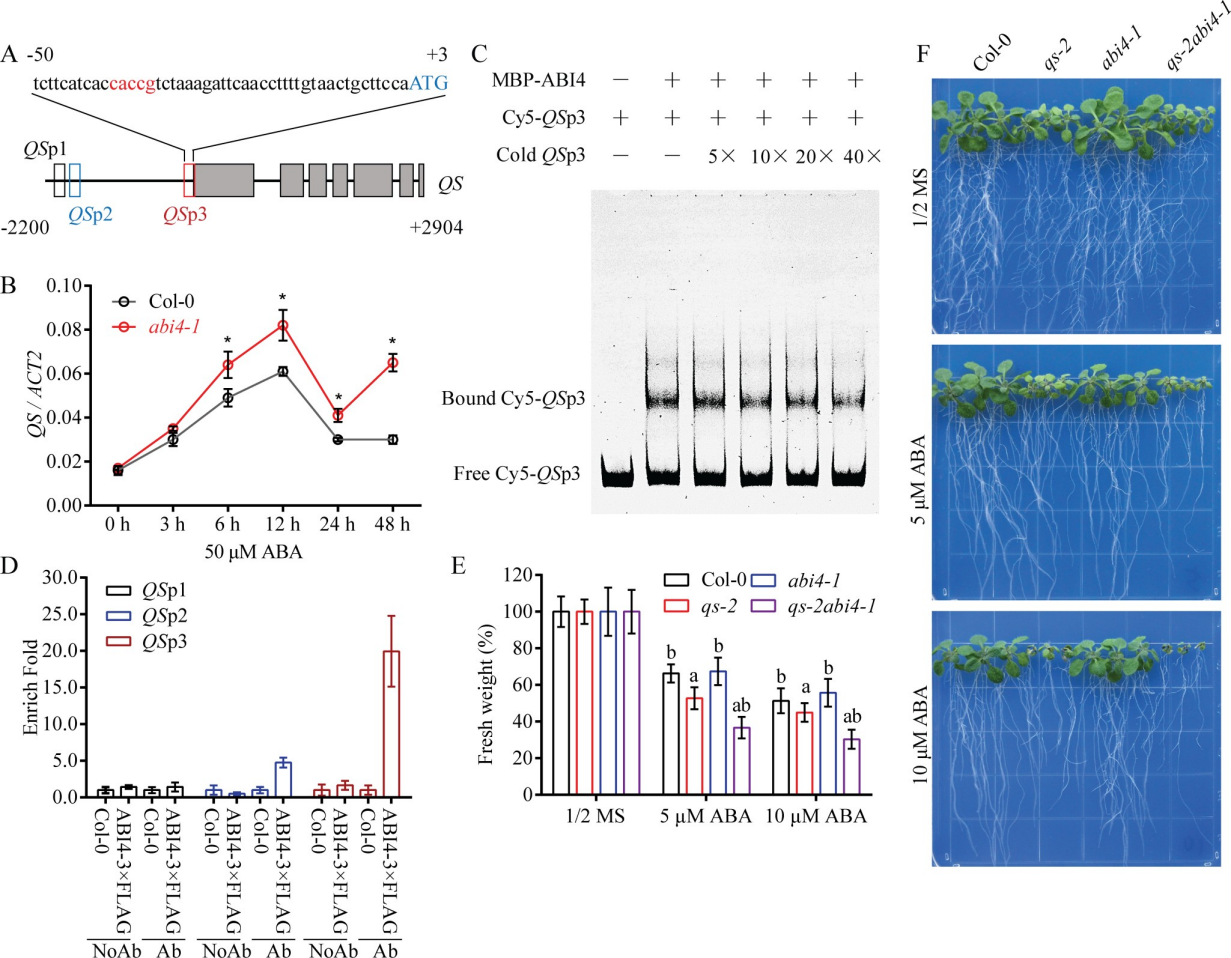
ABI4 negatively regulates *QS* gene expression. (A) Schematic diagram of the *QS* gene. The predicted ABI4-binding sequence CE1 motif is highlighted in red. The gray boxes denote the exons. The red box indicates the predicted ABI4-binding region with the CE1 motif. The black and blue boxes are the *QS* promoter regions used as controls in the assay. (B) The transcript levels of *QS* in Col-0 and *abi4-1* in response to ABA treatment. Ten-day-old seedlings were subjected to 50 μM ABA treatment for 0, 3, 6, 12, 24 or 48 h. The values represent the means ±SD (n = 3 repeats). * *P* < 0.05, Student’s t-test. *ACT2* was used as an internal control. (C) Electrophoretic mobility shift assay showing the binding activity of the recombinant ABI4 protein to the *QS* promoter. The assay was performed using the indicated Cy5-labeled probes and 5×, 10×, 20× or 40× unlabeled competitors. (D) ChIP assay of the binding of ABI4 to different promoter regions of *QS*. The chromatin was extracted from 12-day-old seedlings of *35S*:*ABI4-3×FLAG* transgenic plants and Col-0. Relative enrichment was calculated as the value of the amplified signal normalized against that of the input. Error bars indicate ± SD of three replicates. (E) Genetic relationship between *QS* and *ABI4*. Five-day-old seedlings of Col-0, *qs-2*, *abi4-1*, and *qs-2abi4-1* grown on 1/2 MS medium supplemented with 0, 5 or 10 μM ABA for 8 days. (F) The fresh weight of the seedlings shown in (E). Values are means ± SD of 3 replicates, and each replicate included 9 plants per genotype. The letters a and b above the columns indicate significant difference relative to Col-0 and *qs-2* mutant, respectively (*P* < 0.05, Student’s t-test).

Through bioinformatics analysis of the promoter region of the *QS* gene, we found an ABI4 binding CE1 motif (CACCG) near the ATG start codon (Fig 7A). The transcript level of the QS gene was induced by ABA treatment, and the induction was significantly enhanced in the *abi4-1* mutant (Fig 7B). This result indicates that ABI4 represses the expression of *QS*, possibly through binding to the *QS* promoter. We tested the binding of ABI4 to the *QS* promoter by using purified recombinant MBP-ABI4 fusion protein and electrophoretic mobility shift assay (EMSA). Reciprocal competitive EMSA demonstrated that ABI4 binds *in vitro* to the *QS* p3 promoter region harboring the CE1 motif (Fig 7C). To verify this binding in plants, we performed a chromatin immunoprecipitation (ChIP) assay with chromatin extracts from the wild type and the *35S*:*ABI4-3×FLAG* transgenic plants treated with ABA for 2 days. ChIP-qPCR results showed that the *QS* p3 fragment containing the CE1 motif was markedly enriched by its association with the ABI4-3×FLAG fusion protein (Fig 6D). These results clearly indicate that ABI4 binds to the *QS* promoter. The *qs-2abi4-1* double mutant was generated and used to determine the genetic relationship between *QS* and *ABI4* in response to ABA. The *qs-2abi4-1* double mutant exhibited a phenotype similar to the *qs-2* single mutant in response to ABA treatment (Fig 7E and 7F), suggesting that *ABI4* functions upstream of *QS* in ABA response.

## Discussion

In *Arabidopsis*, targeted screening for phenotypes from specific pools of T-DNA insertion mutants has proven to be an efficient way to identify the functions of a specific type of proteins in biological processes including abiotic stress responses [47–49]. In this study, we carried out a genetic screen for chilling sensitive mutants from a pool of 510 *Arabidopsis* mutants with T-DNA insertions in genes encoding membrane proteins. A chilling hypersensitive mutant was isolated and designed as *htc1* which harbors a T-DNA insertion in the *HIR2* gene. Subsequently analysis indicated that the mutation in the *HIR2* gene is not the causal gene for the phenotype (Fig 1A). By using map-based cloning and genome resequencing, we identified a C1201G mutation in the coding region of the *QS* gene as responsible for the chilling hypersensitivity of the *htc1* mutant, which was renamed as *qs-2* (Fig 1B and S1 Table). The *qs-2* mutation results in an amino acid substitution of Q288E in the NadA domain of the chloroplast localized QS enzyme (Fig 2F and 2G) that catalyzes a critical step of NAD biosynthesis [4]. Since the homozygous null alleles of *qs* mutants are embryo-lethal [40], the *qs-2* allele is likely a weak allele with a partial loss-of-function of the QS enzyme, resulting in reduced NAD biosynthesis and accumulation (Fig 2I).

NAD biosynthesis and metabolism must be finely regulated to maintain the physiological functions of plant cells. NAD is synthesized in higher plants via two biosynthetic pathways: the *de novo* biosynthesis that starts from the oxidation of L-aspartate in the plastid and the salvage pathway consisting of a metabolic cycle in the cytosol. The *de novo* biosynthetic pathway consists of three enzymes, AO, QS, and QPT, that catalyze the early steps of NAD biosynthesis in the plastid, which is the primary route for the production of NAD in plant cells [33]. NAD is a key link between energy metabolism and redox reactions and has emerged as a central hub between bioenergetics and all major cellular processes in plants and other organisms [3]. Redox reactions involving electron or energy transfer to oxygen produce ROS under normal and particularly abiotic stress conditions [46,50]. Therefore, disruption of NAD homeostasis is likely to affect ROS accumulation, plant growth and acclimation to stressful environmental conditions [4,51]. The *qs-2* mutant produces a reduced level of NAD and accumulates excessive ROS (Figs 2I and 6A), which might account for the phenotypes of hypersensitivity to salt stress and ABA, and drought resistance (Fig 3). Our results imply that the steady state of NAD plays a critical role in ROS accumulation and plant response to multiple abiotic stresses. A recent study showed that NAD regulates stress-induced accumulation of ABA and proline and plays an important role in salt stress response in *Arabidopsis* [42]. We found in this study that the *qs-2* mutant accumulates a lower level of ABA, which is rescued by supplementation with NAD precursors (S3A Fig). Our results indicate that the reduced ABA level is not the cause of ABA-hypersensitivity of the *qs-2* mutant since the *aba2-1* mutant, which has only about 30% of the endogenous ABA in wild type [12], is not hypersensitive to ABA (S3B and S3C Fig). Therefore, the ABA-hypersensitivity caused by reduced NAD is likely mediated by the over-accumulated ROS.

In *Arabidopsis*, SnRK2.2, SnRK2.3, and SnRK2.6 play a central role in the ABA signaling pathway, and the phosphorylation of RBOHF by these ABA-activated SnRK2 kinases is required for apoplastic ROS production in response to ABA [21,52]. In this study, we showed that the ABA-hypersensitivity in seedling growth and H_2_O_2_ over-accumulation of the *qs-2* mutant can be completely restored by the *snrk2*.*2/2*.*3/2*.*6* triple mutations (Figs 5 and 6B). This result strongly supports that the ABA signaling component SnRK2s mediate the ABA-hypersensitivity and ROS over-accumulation of the *qs-2* mutant. Furthermore, we found that the ABA-hypersensitivity and H_2_O_2_ over-accumulation of *qs-2* are suppressed by the *rbohF3* mutation (Fig 6C and 6E), indicating the SnRK2-activated RBOHF is the source of ROS generation in the *qs-2* mutant. However, how NAD and/or NAD/NADH ratio modulates SnRK2 activity is currently unknown. It is possible that, like the mammalian AMP-activated protein kinase (AMPK), the plant SnRK2s, belonging to the SNF1/AMPK family, could also be activated by energy deficit with lower NAD and environmental stress conditions [53]. Modulation of SnRK2s activity by NAD may be involved in energy metabolism and energy redistribution in plant response to stress conditions. Alternatively, modulation of SnRK2s activity by NAD could be mediated through PP2Cs. The NAD steady state is necessary for the maintenance of cellular redox balance and scavenging ROS [54]. Under stress conditions, NAD biosynthesis is compromised resulting in ROS accumulation. High cellular ROS inhibits PP2Cs and thus leading to the activation of SnRK2s and ABA responses [20]. Nonetheless, these interesting but highly speculative mechanisms of NAD-mediated SnRK2s modulation requires further experimental verification in the future.

As a precursor, NA is important in NAD metabolism. NA can be decorated by *N*-methylnicotinate, *N*-glucoside, *O*-glucoside, and methyl ester, and these conjugation processes function in the detoxification of NA in plant cells [36,37,55]. Among these conjugates, methyl nicotinate (MeNA) is conducive to NAD production and this conjugation is catalyzed by the nicotinate methyltransferase (NaMT1), while the methyl group is removed from MeNA by methylesterase 2 (MES2) in *Arabidopsis*. High ABA and salt treatment enhance *NaMT1* transcript levels and decrease the transcript level of *MES2* [40,55], which suggests that the NAD salvage pathway is regulated by abiotic stresses in plants. Our results showed that ABI4, an ERF/AP2 transcription factor involved in ABA response, directly binds the promoter region of *QS* gene to repress its expression (Fig 7A–7D), suggesting that the NAD *de novo* biosynthesis is also modulated through the ABA signaling pathway. The ABA-hypersensitive phenotype of *qs-2* mutant is restored by supplementing NA or NaMN (Fig 4), supporting that ABA response is regulated by NAD levels in plants. Our results provide strong links between ABA signaling and NAD biosynthesis.

We propose a model integrating NAD biosynthesis, ABA signaling and ROS production, which is illustrated in S5 Fig. Five enzymatic reactions are responsible for the biosynthesis of NAD from L-Aspartate. The first three enzymes, including AO, QS, and QPT in the *de novo* pathway, function in plastids. The expression of *QS* is repressed by ABI4, which is phosphorylated and activated by three SnRK2 kinases. The NAD salvage pathway starts from NAM, which is sequentially catalyzed by nicotinamidase (NIC1), nicotinate phosphoribosyltransferase (NaPRT), nicotinamide mononucleotide adenylyltransferase (NaMNAT), and NAD synthase (NADS) in the cytoplasm. NaMN is converted to NAD via adenylation by NaMNAT, followed by amidation via NADS. In the *qs-2* mutant, the ROS accumulation is dependent on the SnRK2s activated RBOHF to catalyze the oxidation of NAD(P)H and the reduction of oxygen. As core components in ABA signaling, the three SnRK2 kinases are required for the ROS production as a result of a reduced level of NAD in the *qs-2* mutant. The important role of NAD-mediated ROS regulation in plant growth control and stress resistance implies that modulation of cellular NAD levels is important for plant growth and response to environmental stress.

## Materials and methods

### Plant materials and growth conditions

The *Arabidopsis thaliana* genetic materials used in this study are in the Columbia-0 background. The T-DNA insertion mutants, *hir2-1* (SALK_092306) and *hir2-4* (SAIL_1274_A05) were obtained from the ABRC. Other mutants used in this study, including *snrk2*.*6*/*ost1-3* (SALK_008068), *snrk2*.*2/2*.*3* (*snrk2*.*2* (GABI-Kat 807G04), *snrk2*.*3* (SALK_107315)), *snrk2*.*2/2*.*3/2*.*6*, *abi4-1*, *aba2-1*, were as described in our previous studies [52,56]. The *rbohF3* was kindly provided by Dr. Zhaojun Ding of Shandong University. After genetic crosses, the homozygous double mutants were identified by PCR-based genotyping and Sanger sequencing. For seedlings in Petri dishes, seeds were surface-sterilized and stored in sterile water at 4°C for 2 days, and the seeds were then sown on half strength Murashige and Skoog (1/2 MS) medium (pH 5.8) containing 1% (w/v) sucrose and 0.6% or 1.2% (w/v) agar. For plants in soil, 7-day-old seedlings grown in agar plates were transplanted to soil and grown in a growth room at 22°C with 16 h light / 8 h dark, unless specified otherwise.

### Map-based cloning and genome resequencing

To clone the gene responsible for the phenotype of *htc1*, the original *htc1* mutant was crossed with *Landsberg erecta* (Ler) to obtain a F2 population for genetic mapping. The *htc1* mutation was first mapped to chromosome 5 between the BAC clone K21P3-57k and MDK4-33k by using simple sequence length polymorphism (SSLP) markers, and the locus was then narrowed down to the region between MPF21-10k and MXI22-56k. For genome resequencing, the genomic DNA was extracted from the *htc1* mutant and whole genome sequencing was carried out by using an illumine HiSeq ×10 platform with paired-end 150 bp reads. DNA sequence variations in the *htc1* mutant were identified after alignment with the wild-type (Col-0) sequence, and a C to G substitution within the mapped region was identified in the second exon of AT5G50210. The T-DNA insertion mutation *hir2-2* and the C1201G substitution, which was named as *qs-2* mutation, were segregated from the original *htc1* mutant and verified by PCR-based genotyping and DNA sequencing.

### Molecular complementation assay

To perform the molecular complementation of the *qs-2* mutant, the genomic region containing the *QS* gene with its ~2,000 bp promoter region was amplified from Col-0 genomic DNA and cloned into the pCambia1305-3×FLAG vector to generate the *QSpro*:*QS-3×FLAG* construct. The *QSpro*:*QS-3×FLAG* construct was introduced into the *qs-2* mutant by using the *Agrobacterium*-mediated floral dip method [57]. The T3 homozygous transgenic plants were used for further analysis.

### Subcellular localization

To determine the subcellular localization of QS protein, the genomic region containing the *QS* gene with ~2,000 bp native promoter was amplified from genomic DNA of Col-0 and *qs-2*, and then cloned into pCambia1300-YFP vector to generate *QSpro*:*QS-YFP* and *QSpro*:*qs-2-YFP* constructs, respectively. These constructs were introduced into Col-0 through *Agrobacterium*-mediated floral dipping. Seven-day-old seedlings of three independent T3 transgenic lines were used to detect florescence signals using a confocal laser-scanning microscope (ZEISS LSM880).

### Histochemical staining

Hydrogen peroxide was detected by using DAB staining as previously described [48]. Twenty-day-old plants were harvested and stained in a dye buffer (0.1 M potassium phosphate buffer, pH 7.0, 0.1% (v/v) Triton X-100) containing 1 mg/mL DAB (Sangon Biotech) for 24 h, and then fixed with a solution (3:1:1 v/v/v ethanol: lactic acid: glycerol) before being photographed.

Detection of GUS activity was performed as previously described [48]. About 2,000 bp native promoter of the *QS* gene was amplified from Col-0 genomic DNA and cloned into pMDC162 vector to generate *QSpro*:*QS-GUS* construct. The construct was introduced to Col-0 using *Agrobacterium*-mediated floral dip method. The transgenic plants were incubated in the staining solution (0.5 mg/mL X-Gluc, 0.1 M potassium phosphate buffer, pH 7.0, 1 mM ferrocyanide, 1 mM ferricyanide, and 0.1% (v/v) Triton X-100) at 37°C for 2 to 24 hours. The chlorophyll of the stained tissues was removed using 70% (v/v) ethanol before photographing. The DAB and GUS images were captured using the Olympus DP72 microscope. The DAB staining intensity was determined by the ImageJ (version 1.50i).

### Physiological assays

For analysis of ABA sensitivity, 5-day-old seedlings grown in 1/2 MS plates were transferred to media containing different concentrations of ABA with or without the NAD precursors. For salt tolerance assay, 5-day-old seedlings were transferred to 1/2 MS medium containing 0, 60 or 100 mM NaCl. After 8 days, the fresh weight was recorded and calculated as a percentage of the control. The experiment was performed three times, and each replicate included nine plants per genotype. For drought resistance assay, 21-day-old plants grown in a growth room at 22°C with 10 h light / 14 h dark were subjected to drought treatment by withholding water for 10 days, and then re-watered for 3 days. The survival rates were analyzed after re-watering for 1 week. Three replicates were performed, and each replicate contained 12 plants per genotype.

### Determination of the contents of chlorophyll, ABA, NAD and NAD-related metabolites

For the analysis of chlorophyll content, the chlorophyll was extracted from 10-day-old seedlings using 80% (v/v) acetone. The supernatant was collected by centrifugation at 12,000 g for 6 min at 4°C, and then the absorption at 645 and 663 nm was detected using a NanoDrop 2000C spectrophotometer (ThermoFisher Scientific). To determine the ABA content, 5-day-old seedlings grown in 1/2 MS plates were transferred to 1/2 MS medium containing 50 μM NA or 50 μM NaMN for additional 8 days, then the samples were collected and ABA was extracted as previously described [58]. The ABA-d6 (Olchemim, Olomouc, Czech Republic) was added to the extracts as an internal standard. ABA content in a 50 mL dilution of each sample was determined using the UPLC-Triple TOF 5600+ system (Sciex, Concord, Canada). To determine the contents of endogenous NAD and the related metabolites, including NaMN, NA, Tg, NAOG and NANG, the rosette leaves of 21-day-old plants grown on 1/2 MS plates were collected, and the metabolites were analyzed using UPLC-QQQ–MS/MS as described [37,40]. To determine NAD/NADH ratio, the NAD and NADH were extracted and measured using a NAD(H) content assay kit (Suobio, China) following the protocol described previously [59].

### Gene expression analysis

To analyze the expression pattern of the *QS* gene, different tissues were collected from wild-type (Col-0), including 12-day-old seedlings, shoot and root of 21-day-old seedlings, seeds, leaves, stems, flowers and siliques of 6-week-old plants for total RNA extraction. For gene expression in response to ABA treatment, 10-day-old seedlings were transferred to a liquid medium containing 50 μM ABA for the indicated hours. Total RNA was extracted with TRIzol reagent (Invitrogen), and cDNAs were synthesized using the iScript cDNA synthesis kit (*BIO-RAD*). All quantitative real-time PCR analyses in this study were performed in a CFX96 Real-time system (*BIO-RAD*) using the ChamQ SYBR qPCR Master Mix (Vazyme Biotech co.,ltd) following the manufacturer’s protocol. Each analysis included at least three replicates. *UBQ5* and *ACT2* were used as internal controls. The primers are listed in the S2 Table.

### Electrophoretic Mobility Shift Assay (EMSA)

The purification of recombinant proteins was carried out as described previously [60]. The full-length CDS of the *ABI4* gene was cloned into the pMAL-c5x vector. The construct was transformed into *E*. *coli* BL21 (DE3) to express the recombinant MBP-ABI4 fusion proteins. The CE1 element was identified by analyzing the promoter of *QS* gene using PlantCARE database [61]. EMSA was performed as previously described [2] with a slight modification. Briefly, the binding ability of ABI4 to the CE1 element in the promoter region of *QS* gene was determined using Cy5-labeled CE1 element-containing DNA fragments mixed with 2 mg of the fusion protein in binding buffer (20 mM Tris (pH 7.9), 5% (v/v) glycerol, 200 mM MgCl_2_, 0.1 M DTT, 4% (w/v) BSA, and 0.5% (w/v) salmon sperm DNA). The mixtures supplemented with increasing amounts (1:10 to 1:40 mass ratios) of unlabeled DNA fragments were used for competitive binding tests. After electrophoresis using native polyacrylamide gels, the Cy5 signals were detected with Starion FLA-9000 (FujiFilm, Japan).

### ChIP assay

Chromatin immunoprecipitation (ChIP) assays were performed in *Arabidopsis* as described previously [62]. Ten-day-old seedling of Col-0 and *35S*:*ABI4-3×FLAG* plants were used. Precipitated chromatin DNA was analyzed using quantitative real-time PCR (qPCR ChamQ SYBR qPCR Master Mix, Vazyme Biotech co.,ltd). The sequences of primers used in this experiment are listed in S2 Table.

### Accession Numbers

Sequence data from this study can be found in The Arabidopsis Information Resource (http://www.arabidopsis.org/) under the following accession numbers: *QS*, AT5G50210*; SnRK2*.*2*, AT3G5050*0*; *SnRK2*.*3*, AT5G66880; *OST1*/*SnRK2*.*6*, AT4G33950; *RBOHF*, AT1G64060; *ABI4*, AT2G40220; *ABA2*, AT1G52340; *ACT2*, AT3G18780; *UBQ5*, AT3G62250.

## Supporting information

S1 Fig Identification and characterization of the *qs-2* mutant and complementation lines. (A) Sanger sequencing to verify the mutation of C1201G in the *qs-2* mutant. (B) The transcript levels of *QS* in 2-week-old seedlings of Col-0 and *qs-2* mutant determined by qRT-PCR. Values are means ± SD (n = 6). (C) Immunoblot analysis of the QS-3×FLAG protein in Col-0 wild-type, *qs-2*, and two complementation lines Com-1 and Com-2. The Coomassie Blue (CCB) staining indicates equal loading of the total proteins. (D) The growth phenotype of 10-day-old seedling of Col-0 wild-type, *qs-2*, Com-1 and Com-2. Bar, 1 cm. (E—G) Analysis of the contents of chlorophyll a (E), chlorophyll b (F) and total chlorophyll (G) in the leaves of 10-day-old Col-0, *qs-2*, Com-1 and Com-2. The letters a and b above the columns indicate significant difference relative to Col-0 and *qs-2* mutant, respectively (*P* < 0.05, Student’s t-test).
(PDF)

S2 Fig Pyridine nucleotide contents in *qs-2* and wild type plants. (A—C) The contents and ratio of NAD and NADH in Col-0 and *qs-2* plants. The contents of NAD (A) and NADH (B) were determined in the seedlings of Col-0 and *qs-2* plants grown in 1/2 MS for 2 weeks. The ratio of NAD and NADH (C) was then calculated based on the values shown in (A) and (B). The values shown are means ± SD (n = 12). Asterisks indicate significant differences between *qs-2* and Col-0 seedlings, *** *P* < 0.001, Student’s t-test.
(PDF)

S3 Fig The genetic relationship between *QS* and *ABA2*. (A) The ABA contents in Col-0 wild-type and *qs-2* plants determined by LC-MS. Five-day-old seedlings of Col-0 and *qs-2* grown on 1/2 MS medium supplemented with 50 μM NaMN or 50 μM NA for 8 days. (B) Genetic relationship between *QS* and *ABA2*. Five-day-old Col-0, *qs-2*, *aba2-1*, *qs-2aba2-1* grown on 1/2 MS supplemented with 0, 5 or 10 μM ABA for 8 days. (C) The relative fresh weight of the seedlings shown in (B). Values are means ± SD of 3 replicates, and each replicate contained 9 plants per genotype. The letters a and b above the columns indicate significant difference relative to Col-0 and *qs-2* mutant, respectively (*P* < 0.05, Student’s t-test).
(PDF)

S4 Fig Identification of the *qs-2snrk2*.*2/2*.*3*, *qs-2snrk2*.*6* and *qs-2snrk2*.*2/2*.*3/2*.*6* mutants. (A—C) Genotyping of the homozygous *qs-2snrk2*.*2/2*.*3* triple mutant (A), *qs-2snrk2*.*6* double mutant (B) and *qs-2snrk2*.*2/2*.*3/2*.*6* quadruple mutant (C). The verification of *qs-2* mutation was performed by Sanger sequencing. LP, left primer; RP, right primer; LBb1.3 or LBo8409, primers of the T-DNA left border.
(PDF)

S5 Fig A model showing the reciprocal regulation between NAD metabolism and ABA response. The *de novo* biosynthesis of NAD starts from L-aspartate in chloroplast. The NaMN serves as an intermediate to activate the salvage pathway in the cytosol. NA plays an important role in maintaining the steady state of NAD. Disruption of NAD biosynthesis in the *qs-2* mutant results in ABA-hypersensitivity, which is mediated by SnRK2.2, SnRK2.3 and SnRK2.6. These SnRK2s enhance ROS production through the activation of RBOHF, leading to over-accumulation of ROS that impacts ABA and stress responses. These kinases also phosphorylate ABI4, which is a transcription factor that binds to the promoter region of *QS* and represses *QS* expression.
(PDF)

S1 Table Identification of the mutations between BAC clones MPF21-10k and MXI22-56k on chromosome 5 in the *htc1* mutant using genome resequencing. (PDF)

S2 Table The sequences of the primers used in this study. (PDF)
