# Supplementary figures and images for: Reciprocal regulation between nicotinamide adenine dinucleotide metabolism and abscisic acid and stress response pathways in Arabidopsis
Source: PLoS Genet. 2020 Jun 22;16(6):e1008892. doi: 10.1371/journal.pgen.1008892 (PMC7332101; doi:10.1371/journal.pgen.1008892)

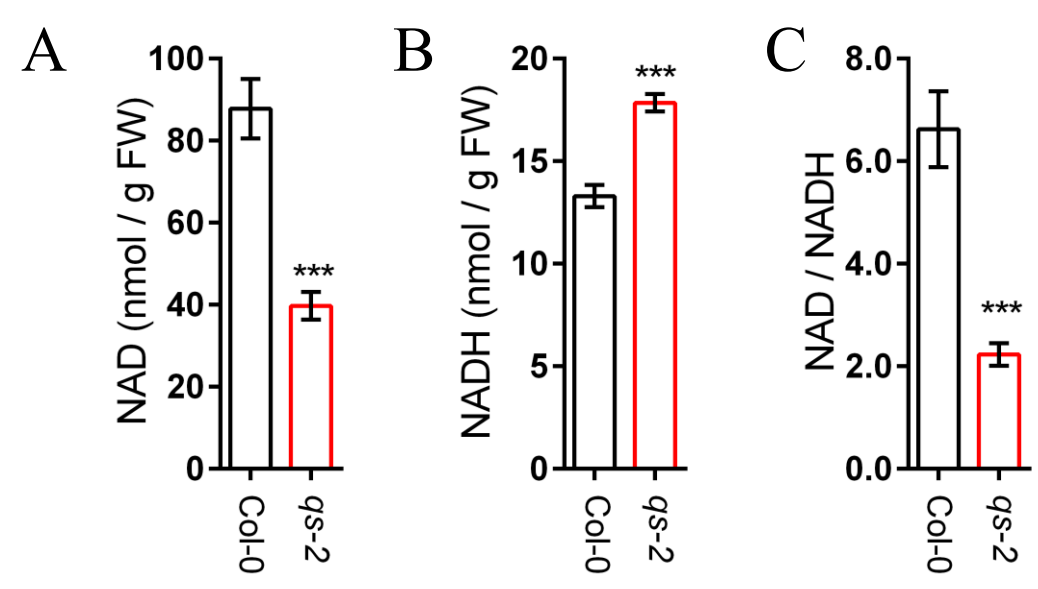

Supplement: S2 Fig — (A—C) The contents and ratio of NAD and NADH in Col-0 and qs-2 plants. The contents of NAD (A) and NADH (B) were determined in the seedlings of Col-0 and qs-2 plants grown in 1/2 MS for 2 weeks. The ratio of NAD and NADH (C) was then calculated based on the values shown in (A) and (B). The values shown are means ± SD (n = 12). Asterisks indicate significant differences between qs-2 and Col-0 seedlings, *** P < 0.001, Student’s t-test. (PDF) [file pgen.1008892.s002.pdf]

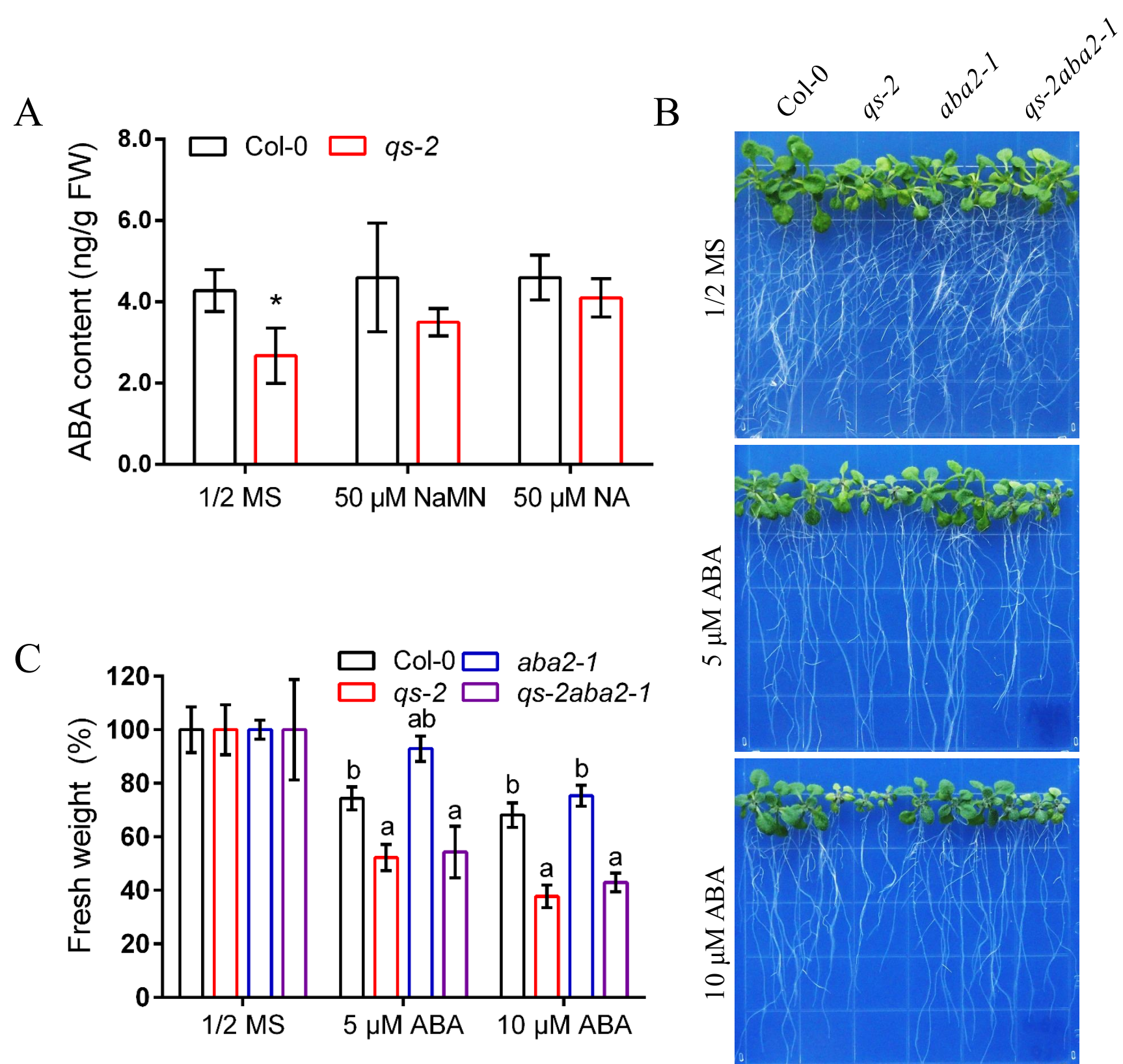

Supplement: S3 Fig — (A) The ABA contents in Col-0 wild-type and qs-2 plants determined by LC-MS. Five-day-old seedlings of Col-0 and qs-2 grown on 1/2 MS medium supplemented with 50 μM NaMN or 50 μM NA for 8 days. (B) Genetic relationship between QS and ABA2. Five-day-old Col-0, qs-2, aba2-1, qs-2aba2-1 grown on 1/2 MS supplemented with 0, 5 or 10 μM ABA for 8 days. (C) The relative fresh weight of the seedlings shown in (B). Values are means ± SD of 3 replicates, and each replicate contained 9 plants per genotype. The letters a and b above the columns indicate significant difference relative to Col-0 and qs-2 mutant, respectively (P < 0.05, Student’s t-test). (PDF) [file pgen.1008892.s003.pdf]

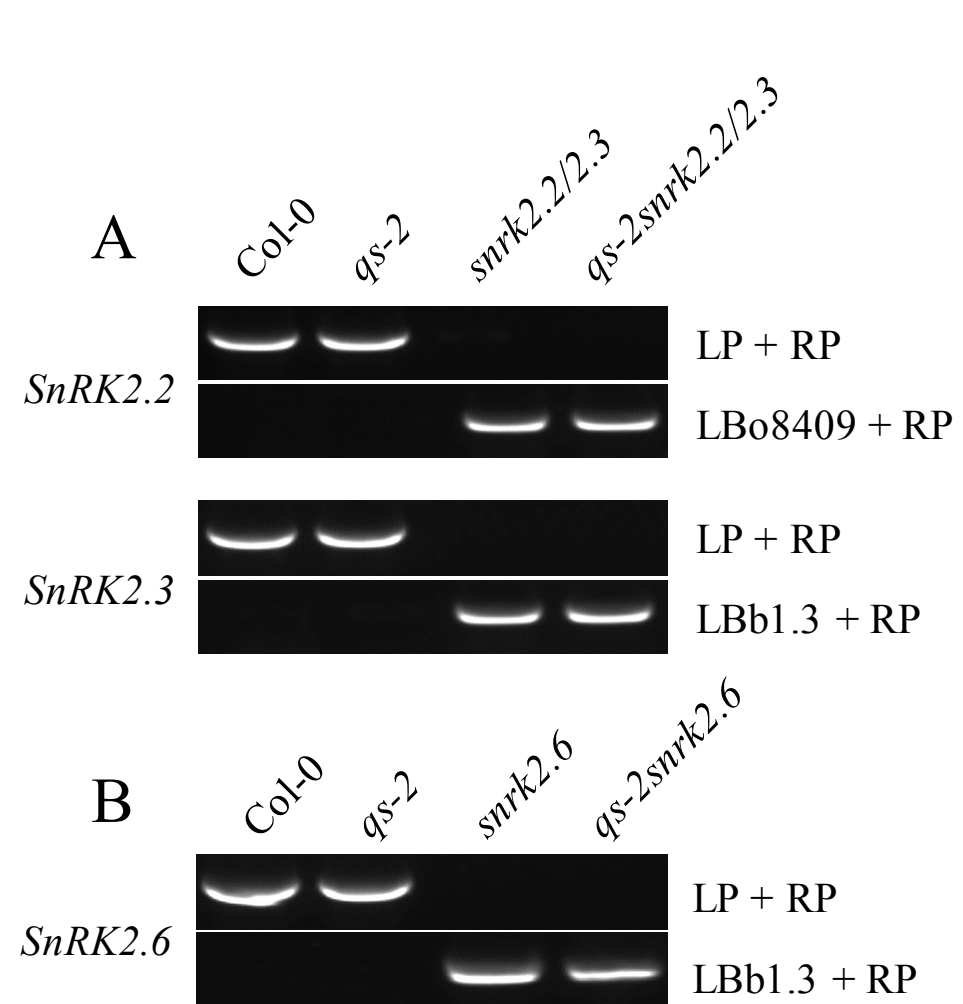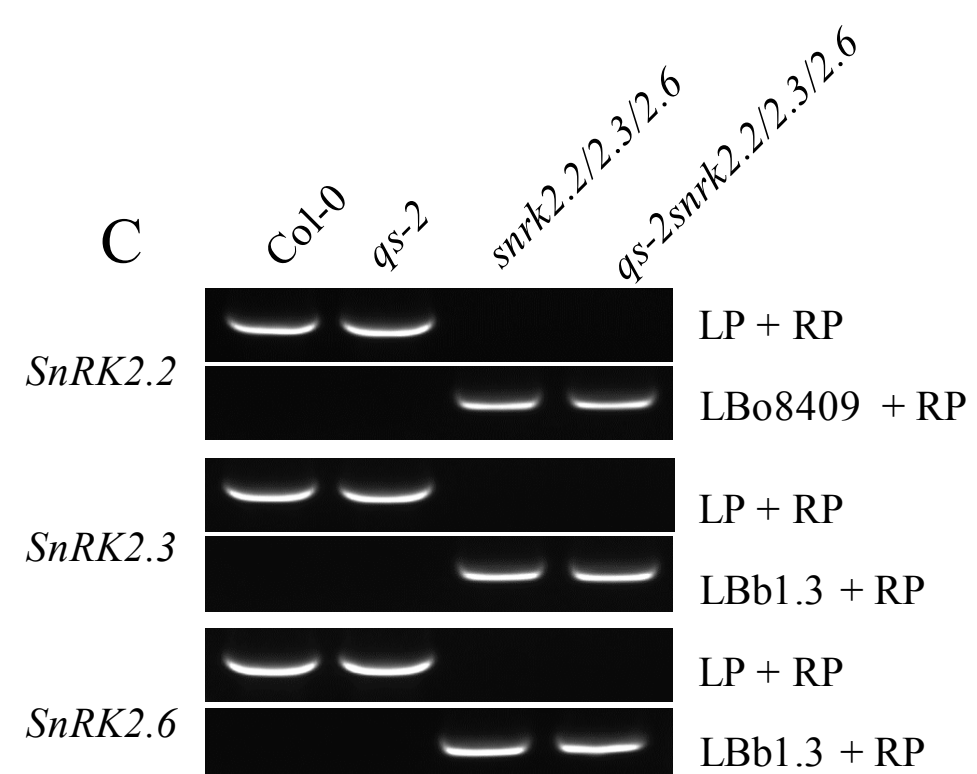

Supplement: S4 Fig — (A—C) Genotyping of the homozygous qs-2snrk2.2/2.3 triple mutant (A), qs-2snrk2.6 double mutant (B) and qs-2snrk2.2/2.3/2.6 quadruple mutant (C). The verification of qs-2 mutation was performed by Sanger sequencing. LP, left primer; RP, right primer; LBb1.3 or LBo8409, primers of the T-DNA left border. (PDF) [file pgen.1008892.s004.pdf]

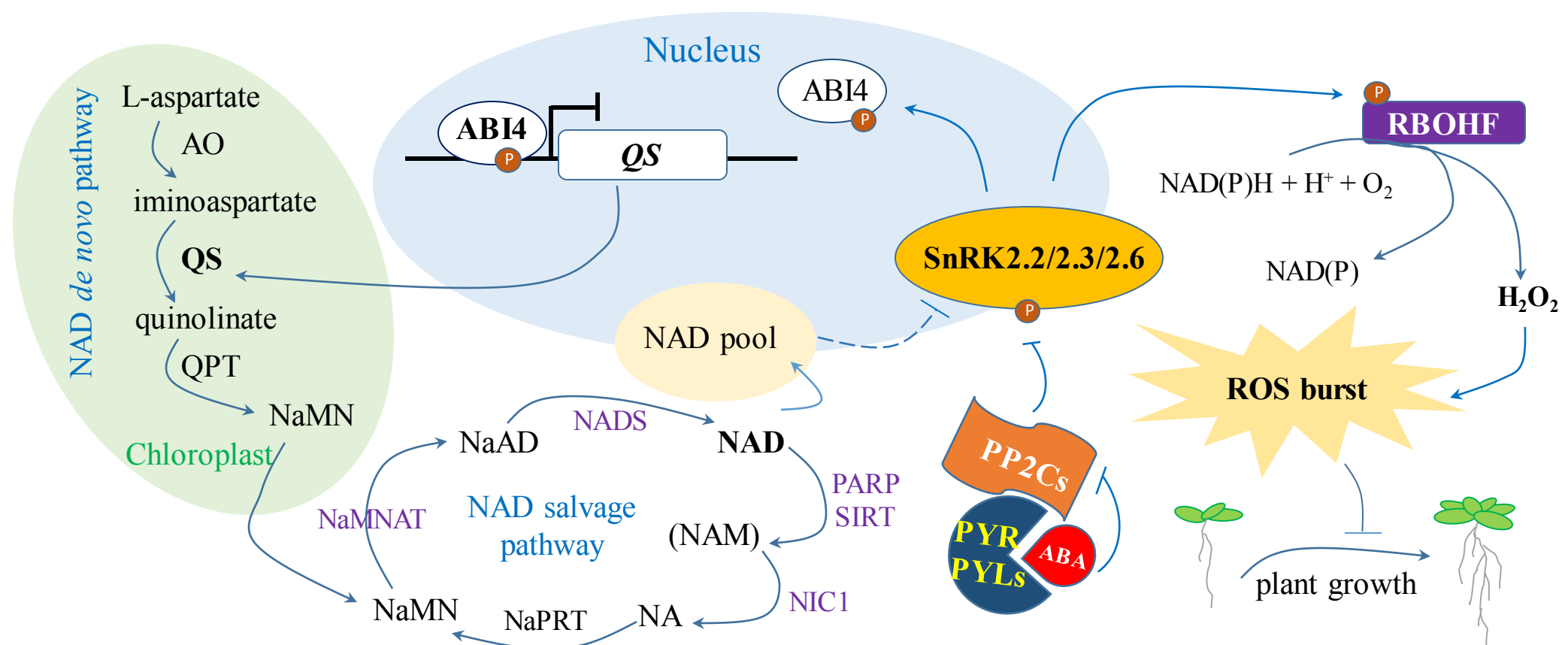

Supplement: S5 Fig — The de novo biosynthesis of NAD starts from L-aspartate in chloroplast. The NaMN serves as an intermediate to activate the salvage pathway in the cytosol. NA plays an important role in maintaining the steady state of NAD. Disruption of NAD biosynthesis in the qs-2 mutant results in ABA-hypersensitivity, which is mediated by SnRK2.2, SnRK2.3 and SnRK2.6. These SnRK2s enhance ROS production through the activation of RBOHF, leading to over-accumulation of ROS that impacts ABA and stress responses. These kinases also phosphorylate ABI4, which is a transcription factor that binds to the promoter region of QS and represses QS expression. (PDF) [file pgen.1008892.s005.pdf]
