# Supplementary material for: Reciprocal regulation between nicotinamide adenine dinucleotide metabolism and abscisic acid and stress response pathways in Arabidopsis
Source: PLoS Genet. 2020 Jun 22;16(6):e1008892. doi: 10.1371/journal.pgen.1008892 (PMC7332101; doi:10.1371/journal.pgen.1008892)
Supplement: S1 Table — (PDF) [file pgen.1008892.s006.pdf]

Chr	Site	Ref	Alt	Region	Gene	Detail	Type	Total-depth	Ref-depth	Alt-depth
Chr5	20359626	-	A	upstream	AT5G50050	43831	_	22	0	22
Chr5	20364893	T	-	intronic	AT5G50070	43831	_	14	0	14
Chr5	20364935	-	A	intronic	AT5G50070	43831	_	11	0	11
Chr5	20444003	C	G	exonic	AT5G50210	c.C862G:p.Q288E	nonsynonymous	31	0	31
Chr5	20921186	-	A	upstream	AT5G51510	43831	_	11	0	11
Chr5	21023424	-	A	downstream	AT5G51750	43831	_	13	1	12
Chr5	21046447	T	A	intergenic	AT5G51795	_	_	14	0	14
Chr5	21175547	-	C	downstream	AT5G52120	43831	_	41	0	41
Chr5	21581896	-	T	upstream	AT5G53200	43831	_	26	0	26
Chr5	21672748	-	A	upstream	AT5G53420	43831	_	7	0	7
