# Supplementary material for: Reciprocal regulation between nicotinamide adenine dinucleotide metabolism and abscisic acid and stress response pathways in Arabidopsis
Source: PLoS Genet. 2020 Jun 22;16(6):e1008892. doi: 10.1371/journal.pgen.1008892 (PMC7332101; doi:10.1371/journal.pgen.1008892)
Supplement: S2 Table — (PDF) [file pgen.1008892.s007.pdf]

Forward Primer	Sequence	Reverse Primer	Sequence	Description
HIR2pg-FP	CTCGAAATGCTGACTGGTAGA	HIR2pg-RP	GGAGGCATTGTTGGCCTGTAA	HIR2pro:HIR2-3×FLAG/Myc
hir2-1-LP	GGGCAACTACTGATCTTTCCC	hir2-1-RP	CGAGCAGAAGAATGAAATTGC	genotyping of hir2-1
hir2-4-LP	CGATTTTTCTCTCGCAAACAG	hir2-4-RP	TCAGCAACTCGATGTTCAGTG	genotyping of hir2-4
QSpro-FP	GGAGATCGATAAGGCTCTGAT	QSpro-RP	TCTCTTGCTCTCACAACTTAATA	Qspro:QS-GUS
QS-qFP	AGGTTGGTGTGTACAGGATGT	QS-qRP	AGCAAACGCCTGCAGAATCG	qRT-PCR
QS-pgFP	GGAGATCGATAAGGCTCTGAT	QS-pgRP	TCTCTTGCTCTCACAACTTAATA	QSpro:QS-3×FLAG
QSpg-FP	GGAGATCGATAAGGCTCTGAT	QSpg-RP	TCTCTTGCTCTCACAACTTAATA	QSpro:QS/qs-2-YFP
RBOHF-qFP	TCGGTTCGACTGCTTAAGGTTGC	RBOHF-qRP	ATGGCGAAACCGCAGGACATTG	qRT-PCR
ACT2-qFP	CGTACAACCGGTATTGTGCT	ACT2-qRP	GATGTCTCTTACAATTTCCCGCT	qRT-PCR
UBQ5-qFP	AG AAG ATC AAG CAC AAG CAT	UBQ5-qRP	CA GAT CAA GCT TCA ACT CCT	qRT-PCR
snrk2.2-LP	TCCTCTGGGATCGAGTATGTG	snrk2.2-RP	TGGTTTAGGTGATTTTGACGC	genotyping of snrk2.2
LBo8409	ATATTGACCATCATACTCATTGC			genotyping of snrk2.2
snrk2.3-LP	TGCTTTTGAGTGCTTTTAATGTG	snrk2.3-RP	ACATCTGCAATCTGGTAACCG	genotyping of snrk2.3
snrk2.6-LP	CATATCTTTAGACGAGGGGCC	snrk2.6-RP	GTGAGTGGTCCAATGGATTTG	genotyping of snrk2.6
rbohF3-LP	CGATCTTCAAGACGATGACAC	rbohF3-RP	GAAGATCTGGAGACGAGAATC	genotyping of rbohF3
dSpm1n	AGTAAGAGTGTGGGGTTTTGG			genotyping of rbohF3
qs-2-seqLP	GGAAGCTTTGCTCAAGCTCAG	qs-2-seqRP	TCTCATCTGACATCCTGTACAC	genotyping of qs-2
ABI4-gFP	ATGGACCCTTTAGCTTCCCAA	ABI4-gRP	ATAGAATTCCCCCAAGATGGG	35S:ABI4:3×FLAG
ABI4-MBP-FP	ATGGACCCTTTAGCTTCCCAA	ABI4-MBP-FP	ATAGAATTCCCCCAAGATGGG	MBP-ABI4
QS-p1-FP	TGCAGTTGACGCAAATGGCAT	QS-p1-RP	TCAGAGCCTTATCGATCTCC	ChIP and EMSA assay
QS-p2-FP	TACCAAGAACTCGAATATCCCA	QS-p2-RP	TCGCTACAATATTGTGACATCG	ChIP and EMSA assay
QS-p3-FP	CAAAGCAAAGTGGAACACTTGC	QS-p3-RP	AGAAGGGTTAGGGGTCCGAG	ChIP and EMSA assay
