# Supplementary material for: Reciprocal regulation between nicotinamide adenine dinucleotide metabolism and abscisic acid and stress response pathways in Arabidopsis
Source: PLoS Genet. 2020 Jun 22;16(6):e1008892. doi: 10.1371/journal.pgen.1008892 (PMC7332101; doi:10.1371/journal.pgen.1008892)
Supplement: S1 Fig — (A) Sanger sequencing to verify the mutation of C1201G in the qs-2 mutant. (B) The transcript levels of QS in 2-week-old seedlings of Col-0 and qs-2 mutant determined by qRT-PCR. Values are means ± SD (n = 6). (C) Immunoblot analysis of the QS-3×FLAG protein in Col-0 wild-type, qs-2, and two complementation lines Com-1 and Com-2. The Coomassie Blue (CCB) staining indicates equal loading of the total proteins. (D) The growth phenotype of 10-day-old seedling of Col-0 wild-type, qs-2, Com-1 and Com-2. Bar, 1 cm. (E—G) Analysis of the contents of chlorophyll a (E), chlorophyll b (F) and total chlorophyll (G) in the leaves of 10-day-old Col-0, qs-2, Com-1 and Com-2. The letters a and b above the columns indicate significant difference relative to Col-0 and qs-2 mutant, respectively (P < 0.05, Student’s t-test). (PDF) [file pgen.1008892.s001.pdf]

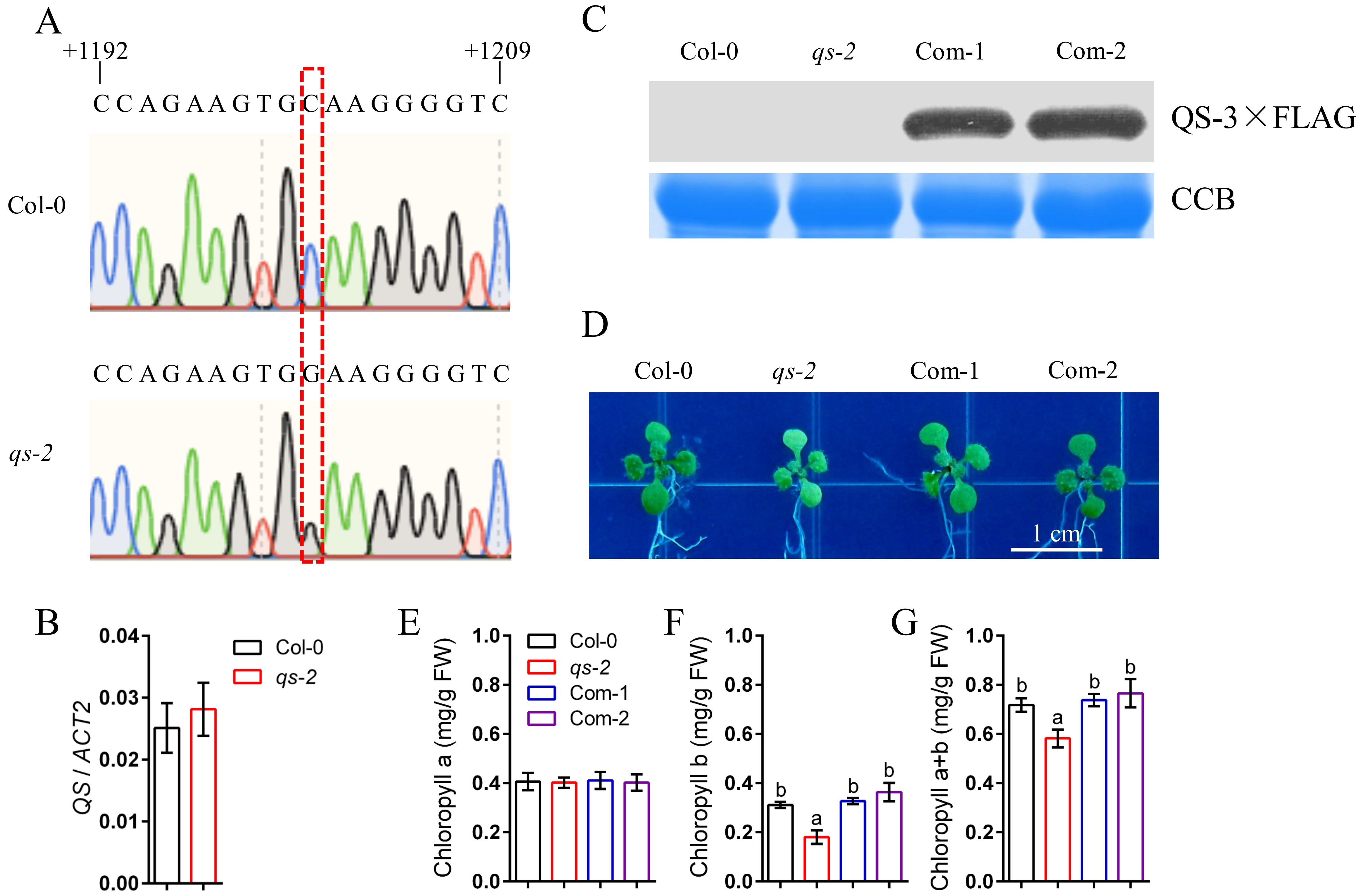
A
+1192
+1209
CCAGAAGTGCAAGGGGTC
Col-0
CCAGAAGTGGAAGGGGTC
qs-2
B
QS / ACT2
0.04
0.03
0.02
0.01
0.00
Col-0
qs-2
C
Col-0
qs-2
Com-1
Com-2
QS-3×FLAG
CCB
D
Col-0
qs-2
Com-1
Com-2
1 cm
E
Chloropyll a (mg/g FW)
1.0
0.8
0.6
0.4
0.2
0.0
Col-0
qs-2
Com-1
Com-2
F
Chloropyll b (mg/g FW)
b
a
b
b
G
Chloropyll a+b (mg/g FW)
b
a
b
b
